# Risk of long COVID main symptoms after SARS-CoV-2 infection: a systematic review and meta-analysis

**DOI:** 10.1038/s41598-023-42321-9

**Published:** 2023-09-15

**Authors:** Zoe Marjenberg, Sean Leng, Carlo Tascini, Megha Garg, Kate Misso, Clotilde El Guerche Seblain, Nabila Shaikh

**Affiliations:** 1 Maverex Ltd, Suite A, 168 Brinkburn Street, The Old Public Library, Newcastle Upon Tyne, NE6 2AR UK; 2 Division of Geriatric Medicine and Gerontology, Department of Medicine, Johns Hopkins Center on Aging and Immune Remodelling, Johns Hopkins University School of Medicine, Baltimore, MD USA; 3 W. Harry Feinstone Department of Molecular Microbiology and Immunology, Johns Hopkins University Bloomberg School of Public Health, Baltimore, MD USA; 4 Infectious Diseases Clinic, Department of Medicine (DAME), Udine University, Udine, Italy; 5 Sanofi, Lyon, France; 6 Sanofi, Reading, UK

**Keywords:** Infectious diseases, Signs and symptoms

## Abstract

This review aimed to summarise the relative risk (RR) of the main symptoms of long COVID in people infected with SARS-CoV-2 compared to uninfected controls, as well as the difference in health-related quality of life (HRQoL) after infection. MEDLINE, EMBASE, PubMed, NLM-LitCovid, WHO-COVID-19, arXiv and Europe-PMC were searched up to 23rd March 2022. Studies reporting risk (four or more weeks after infection) of fatigue, shortness of breath, and cognitive dysfunction, as well as comparative HRQoL outcomes, were included. Pairwise random-effects meta-analyses were performed to pool risks of individual symptoms. Thirty-three studies were identified; twenty studies reporting symptom risks were included in the meta-analyses. Overall, infection with SARS-CoV-2 carried significantly higher risk of fatigue (RR 1.72, 95% confidence intervals [CIs] 1.41, 2.10), shortness of breath (RR 2.60, 95% CIs 1.96, 3.44), memory difficulties (RR 2.53, 95% CIs 1.30, 4.93), and concentration difficulties (RR 2.14, 95% CIs 1.25, 3.67). Quality of life findings were varied and comparisons between studies were challenging due to different HRQoL instruments used and study heterogeneity, although studies indicated that severe hospitalised COVID is associated with a significantly poorer HRQoL after infection. These risks are likely to constantly change as vaccines, reinfections, and new variants alter global immunity.

## Introduction

### Background

A growing body of evidence has shown that a significant proportion of COVID-19 survivors experience persistent symptoms after the acute phase of SARS-CoV-2 infection, also known as long COVID^1^. Several terms have been proposed to describe long COVID, however there is a lack of standardised nomenclature or diagnostic criteria. The National Institute for Health and Care Excellence (NICE) in the UK defines ‘long COVID’ as "signs and symptoms that develop during or after an infection consistent with COVID‑19, continue for more than 4 weeks and are not explained by an alternative diagnosis^1^." Under this definition, long COVID consists of two categories, ongoing symptomatic COVID-19, where symptoms last for 4–12 weeks, and post-COVID-19 syndrome, where symptoms persist beyond 12 weeks^2^. According to the World Health Organization (WHO), “post-COVID-19 condition occurs in individuals with a history of probable or confirmed SARS-CoV-2 infection, usually 3 months from the onset of COVID-19 with symptoms that last for at least 2 months and cannot be explained by an alternative diagnosis”^3, 4^.

The number of people globally with long COVID is currently unknown, and so far attempts to estimate prevalence have resulted in heterogenous findings^5^. A recent systematic review and meta-analysis has estimated that there are around 200 million individuals affected by post-COVID-19 syndrome^6^. Three recent meta-analyses have found that fatigue is the most common post-COVID-19 sequelae, while also finding cognitive dysfunction to be common^6–8^. These reviews assessed different length of time post COVID investigating 4+ weeks^6^, 12+ weeks^8^, and 12+ months^7^, respectively. In addition, a recent synthesis of ten longitudinal study samples from the UK found the proportions of presumed COVID-19 cases reporting any symptoms for 12+ weeks after infection ranged from 7.8 to 17%, while 1.2% to 4.8% reported debilitating symptoms^9^.

The risk of developing long COVID, and of its specific symptoms remains unclear. This is further complicated by the fact that many of the symptoms associated with long COVID are also common in the general population. A recent systematic review attempted to assess the risk of post-COVID-19 fatigue and estimated the risk of fatigue to be 3.7 times higher in post-COVID patients than in healthy controls 76–97 days after infection^10^. However, this review only included three studies in the meta-analyses, due to relatively early date that the review searches were performed (February 2021).

### Aim of this review

While a number of systematic reviews and meta-analyses on long COVID symptoms exist, including those characterizing persisting post-COVID-19 symptoms and impact on quality of life^11–13^, these reviews do not assess the risk of these symptoms after infection compared with uninfected people, and therefore the excess risk of post-COVID-19 syndrome is poorly understood.

This review aims to systematically investigate the relative risk of three major long COVID symptoms (fatigue, shortness of breath/dyspnoea, and cognitive dysfunction) and quality of life ≥ 4 weeks from SARS-CoV-2 infection compared to non-infected controls. These three symptoms were selected for study as they are often the most prevalent symptoms of long COVID reported in the literature, and are the three named symptoms in the December 2021 WHO Delphi consensus on the definition of post COVID-19 condition^14^.

## Results

### Literature search

**Figure 1:**
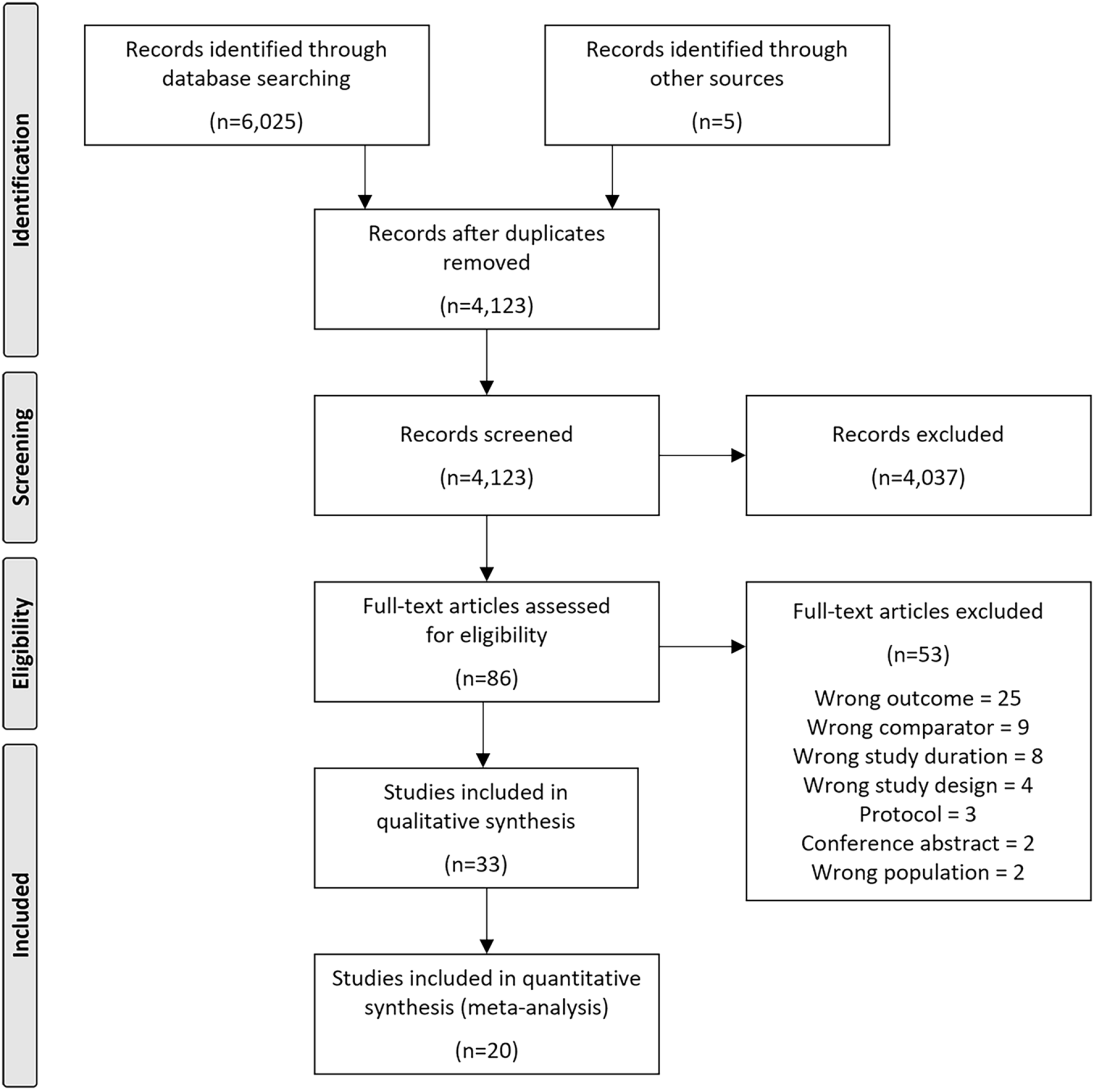
Flow diagram of study selection^67^.

The searches identified 6025 records from electronic databases and five records from hand searching. After removing duplicates, 4123 records were title/abstract screened, of which 86 full-text articles were retrieved and assessed for eligibility. Studies excluded at full-text screening, with reasons for exclusion, are listed in Supplementary Table S3. A total of 33 studies were identified for inclusion in the systematic review (Fig. 1).

### Study and patient characteristics

The characteristics of the 33 included studies are summarised in Table 1, with further details available in Supplementary Table S4. Studies were performed in a total of 15 countries, with the majority from Europe and North America. Outcomes reported by the studies were risk of fatigue^15–34^, shortness of breath/dyspnoea^15–17, 19, 22–25, 27–31, 35, 36^, cognitive dysfunction^15, 17–20, 22–25, 27, 29–31, 33, 37, 38^, and comparisons of quality of life^23, 36, 39–47^.

**Table 1:** Characteristics of included studies.

Study	Country	Data source	Study design	COVID-19 test	COVID + population	N cases/controls
Al-Aly^16^	USA	Veterans’ Health Administration database	Cohort	NR	Adults; military veterans	73,435/4,990,835
Al-Aly^15^	USA	Veterans’ Health Administration database	Cohort	NR	Adults; military veterans	113,474/4,983,491 (COVID) 5,785,273 (influenza)
Amin-Chowdhury^17^	UK	Public Health England database	Cohort	Antibody test	Adults; healthcare workers	140/1160
Borch^18^	Denmark	Danish Health Data Authority and other national registries	Cohort	PCR	Children (0–17 yrs)	15,041/47,780
Carazo^37^	Canada	Provincial COVID-19 and SARS-CoV-2 laboratory databases	Case–control	PCR	Adults; healthcare workers	6052/4390
Caspersen^19^	Norway	Norwegian Mother, Father and Child Cohort Study and linked registries	Cohort	PCR	Adults; participants in MoBa cohort study	774/72,953
Castro^20^	USA	Electronic hospital records	Case–control	PCR	Adults; hospitalised; emergency inpatients	6619/6342
Chevinsky^21^	USA	Premier Healthcare Database Special COVID-19 Release	Matched cohort	NR	Adults; outpatients	44,489/44,489
Desgranges^22^	Switzerland	Hospital patients	Cohort	PCR	Outpatients	418/89
Elkan^39^	Israel	Hospital patients	Case–control	PCR	Adults; hospitalised	66/42
Huang^40^	China	Hospital patients	Cohort	PCR	Hospitalised	1164/1164
Kikkenborg Berg^23^	Denmark	Danish COVID-19 database	Matched cross-sectional	PCR	Adolescents (15–18 yrs)	6630/21,640
Kuodi^24^	Israel	Hospital testing laboratory	Cross-sectional (from cohort study)	PCR	Adults; tested in hospital	951/2437
Liu^38^	China	Hospital patients	Cohort	NR	Adults (≥ 60 yrs); hospitalised	1438/438
Matta^25^	France	French CONSTANCES cohort	Cross-sectional (from cohort study)	Antibody test	Adults (18–69 yrs)	1091/25,732
Nielsen^35^	Denmark	Hospital healthcare workers	Cohort	PCR	Adults, healthcare workers; non-hospitalised	210/630
Niyatiwatchanchai^41^	Thailand	Hospital patients	Matched cross-sectional	PCR	Adults; hospitalised	105/25
Noviello^26^	Italy	Hospital testing laboratory	Cohort	PCR	Adult (18–60 yrs); tested in hospital	164/183
Petersen^36^	Germany	Medical centre clinical information system and community participants	Matched cross-sectional	PCR	Adults (45–74 yrs)	443/1328
Radtke^47^	Switzerland	Proxy-reported questionnaires	Cross-sectional (from cohort study)	Antibody test	Children and adolescents (6–16 yrs)	109/1246
Raman 2020^42^	UK	Hospital patients	Matched cohort	PCR	Adults	58/30
Rivera-Izquierdo^27^	Spain	Hospital patients	Cohort	PCR	Hospitalised	453/453
Roessler^28^	Germany	Health insurance database	Matched cohort	Laboratory virus detection	Adults and children; hospitalised/outpatient	678,965/3,394,825
Roge^29^	Latvia	Hospital patients	Cohort	Rapid antigen test	Children (1 mth-18 yrs), hospitalised/outpatient	236/142
Søras^44^	Norway	Laboratory records	Cohort	PCR	Adults	794/7978
Søras^45^	Norway	Laboratory records	Cohort	PCR	Adults	794/7992
Sørenson^30^	Denmark	National COVID-19 surveillance system	Cross-sectional	PCR	Adults (≥ 15 yrs)	61,002/91,878
Spotnitz^31^	USA	Health insurance database	Cohort	NR	Adults and children; hospitalised	448,176/803,870
Stephenson^46^	UK	Public Health England database	Cohort	PCR	Adolescents (11–17 yrs)	3065/3739
Strahm^32^	Switzerland	Hospital employees	Cohort	PCR/rapid antigen test	Adults; healthcare workers	784/2550
Taquet^33^	USA	TriNetX Analytics	Cohort	PCR	Adolescents and adults (≥ 10 yrs); hospitalised	106,578/106,578
Vlake^43^	Netherlands	ISARIC database and Franciscus Corona Registry	Cohort	PCR	Adults; hospitalised	1446/148
Xie^34^	USA	US Department of Veterans Affairs	Cohort	NR	Adults	181,384/4,397,509

mth, months; NR, not reported; PCR, polymerase chain reaction; yrs, years.

Participants or records in the studies were retrieved or recruited from healthcare databases or registries, COVID-19 databases, hospitals (including inpatients and outpatients, healthcare workers, and hospital employees), COVID-19 laboratory records, questionnaires, existing study cohorts, and schools. Case populations included people with a positive COVID-19 test, users of healthcare facilities with a positive test, volunteers or survey respondents, and hospitalized patients or outpatients of hospitals. Studies were classed by this review as either those including hospitalized patients, hospital outpatients, or those more representative of the general population (all SARS-CoV2 infections). Two studies included individuals who were tested for SARS-CoV-2 infection in hospital but did not clarify whether these were hospitalized patients^24, 26^; these were therefore considered representative of a general population by this review. Control populations included those testing negative for SARS-CoV-2 infection (31 studies) or a historical influenza cohort to compare COVID-19 with a well-characterized respiratory viral illness (four studies). Nearly all studies included controls from the same population as the cases; of the 13 studies including outpatients and hospitalized patients, three studies included non-hospitalized individuals as controls^38, 41, 42^.

Most included studies enrolled adults only. The reported ages of COVID-positive participants in studies that included adults ranged from a median of 39.8 to 70.8 years, and a mean of 35.6 to 60.7 years. Five studies enrolled children or adolescents^18, 23, 29, 46, 47^. The reported percentage of female participants ranged from 5.4 to 84.3%.

SARS-CoV-2 infection was assessed by PCR (22 studies), antibody test (3 studies) or rapid antigen test (2 studies); the remaining studies did not report the diagnostic test used. Only one study reported details concerning the main circulating variant during the time period for SARS-CoV-2 infection^46^. The original wild-type COVID-19 strain was estimated to be in circulation during the infection period in most studies.

### Risks of fatigue, shortness of breath, and cognitive dysfunction after SARS-CoV-2 infection

Risk of post-COVID symptoms ≥ 4 weeks after infection was reported by 20 studies for fatigue (Supplementary Table  S5), 15 studies for shortness of breath (Supplementary Table S6), and 16 studies for cognitive dysfunction (Supplementary Table S7). COVID-19 cases were compared to COVID-19-negative controls in most studies; four studies compared risks in hospitalised COVID-19 patients to hospitalised historical influenza cohorts. While outcome descriptions were similar across fatigue and shortness of breath, cognitive dysfunction outcome descriptions were more varied, and included general cognition outcomes (e.g., cognitive impairment), memory-related problems, concentration-related problems and language disturbances.

At least one significantly higher outcome risk was reported by 15 studies for fatigue, by 11 studies for shortness of breath, and by nine studies for cognitive dysfunction.

Compared to historical hospitalised influenza cohorts, risks of long COVID-19 symptoms ranged from 1.30 (95% CIs not reported) to 2.65 (95% CIs 2.22, 3.08) for fatigue, 1.14 (95% Cis 0.94, 1.40) to 2.28 (95% CIs not reported) for shortness of breath, and from 1.18 (95% Cis 0.89, 1.48) to 1.47 (95% Cis 1.15, 1.87) for cognition.

#### Meta-analyses of risks of fatigue, shortness of breath, and cognitive dysfunction after SARS-CoV-2 infection

Meta-analyses (random-effects) of risks of fatigue, shortness of breath, and cognitive dysfunction after SARS-CoV-2 infection included all studies of adolescents and/or adults comparing COVID-19-positive cases with COVID-19-negative controls and reporting a comparable risk outcome. Comparisons of historical influenza cohorts were not included as of the four studies, two were additional sub-analyses of hospitalised patients, one did not report a comparable risk ratio, and one did not report 95% CIs. Fourteen studies were eligible for inclusion in the meta-analysis for fatigue and 12 for shortness of breath. Cognitive dysfunction outcomes were classified into those reporting general cognition problems (three studies), memory-related problems (seven studies), and concentration-related problems (six studies).

**Figure 2:**
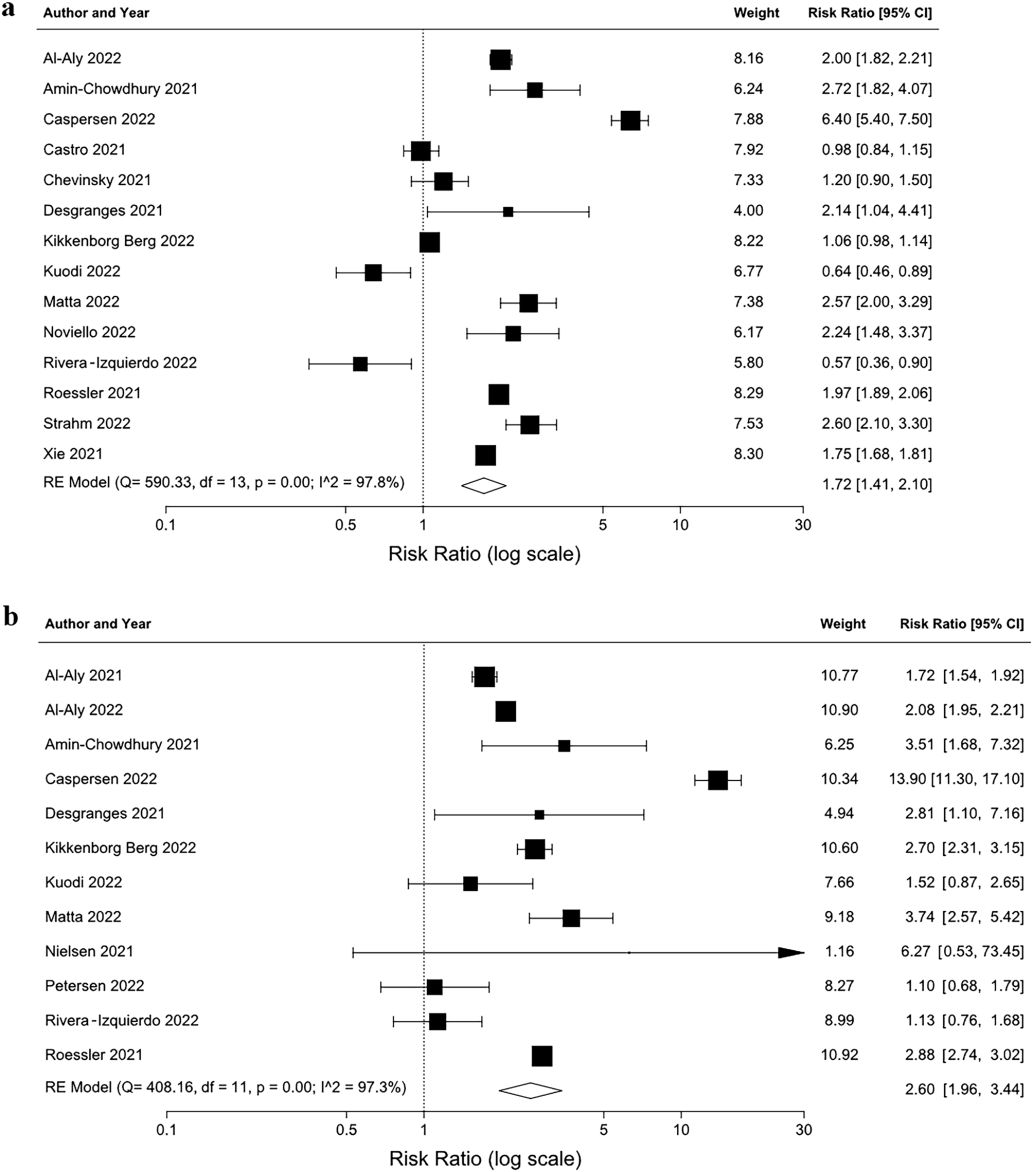
Meta-analyses results (random-effects) of risk of fatigue (**a**) and shortness of breath (**b**) after SARS-CoV-2 infection. Arrow indicates upper 95% CI is greater than risk ratio scale range shown. df, degrees of freedom; RE, random-effects.

Compared to non-infected controls, SARS-CoV-2 infection was associated with a significantly higher risk of fatigue (RR 1.72 [95% CIs 1.41, 2.10]) and shortness of breath (RR 2.60 [95% CIs 1.96, 3.44]) ≥ 4 weeks after the infection (Fig. 2). Analyses of studies or subgroups only reporting risks for hospitalised patients/outpatients found a risk ratio of 1.59 (95% CIs 1.20, 2.11) for fatigue (seven studies) and 2.78 (95% CIs 2.31, 3.34) for shortness of breath (five studies).

**Figure 3:**
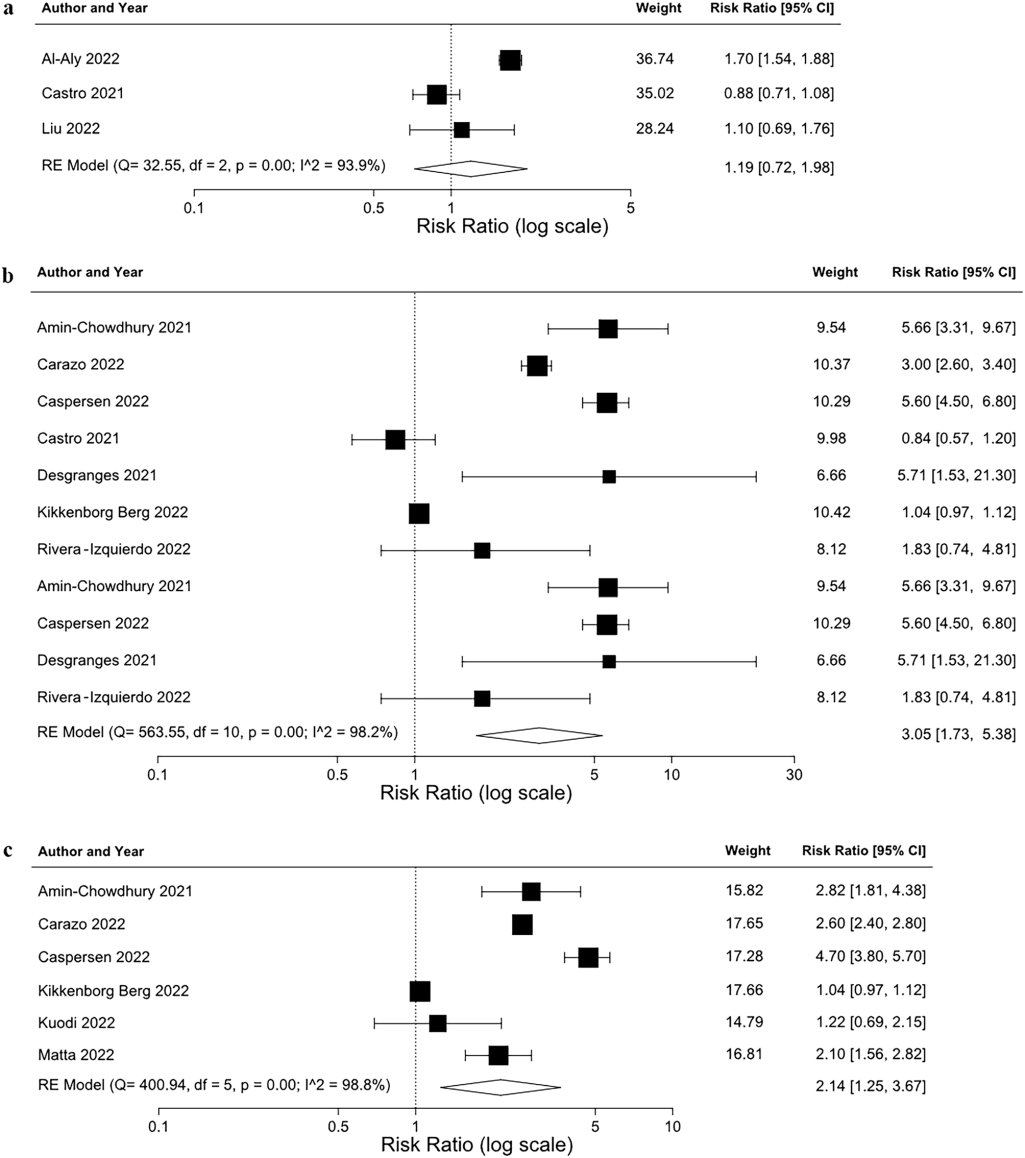
Meta-analyses results (random-effects) of risk of cognitive problems (**a**), memory problems (**b**), and concentration problems (**c**) after SARS-CoV-2 infection. df, degrees of freedom; RE, random-effects.

Both memory and concentration-related problems had a significantly higher risk in COVID-positive participants (memory: RR 2.53 [95% CIs 1.30, 4.93]; concentration: RR 2.14 [95% CIs 1.25, 3.67]) ≥ 4 weeks after infection; while there was an increased risk of cognition problems (RR 1.44 [95% CIs 0.59, 3.56]), this was not statistically significant (Fig. 3).

#### Sensitivity analyses, heterogeneity, leave-1-out analyses, and publication bias

Sensitivity analyses were performed for all four symptoms, where three or more studies could be included in the analysis (Table 2). All fatigue and shortness of breath sensitivity analyses showed a similar result to the main analyses with no loss of statistical significance, indicating that the results were generally robust. Removal of preprints from the memory and concentration analyses, and removal of post-vaccination studies from the concentration analysis, resulted in a loss of significance, however these analyses included a small number of studies.

**Table 2:** Sensitivity analyses.

Sensitivity analysis	Risk ratio (95% CIs)	Q	df	Q *P*-value	I^2^ (%)
**Fatigue**					
≥ 12 weeks after infection	1.75 (1.49, 2.04)	154.71	9	< 0.01	94.2
Included population overlap study	1.73 (1.44, 2.07)	590.45	14	< 0.01	97.6
Included children-only study	1.78 (1.46, 2.17)	596.68	14	< 0.01	97.7
Pre-vaccination studies only	2.05 (1.53, 2.74)	250.41	8	< 0.01	96.8
Adjusted risks only	1.69 (1.30, 2.21)	553.17	12	< 0.01	97.8
Peer-reviewed publications only	1.89 (1.41, 2.54)	468.10	9	< 0.01	98.1
**Shortness of breath**					
≥ 12 weeks after infection	2.45 (1.86, 3.22)	125.51	6	< 0.01	95.2
Included children-only study	2.59 (1.96, 3.42)	408.19	12	< 0.01	97.1
Pre-vaccination studies only	2.83 (1.77, 4.52)	343.65	8	< 0.01	97.7
Adjusted risks only	2.56 (1.73, 3.80)	362.22	10	< 0.01	97.2
Peer-reviewed publications only	2.63 (1.70, 4.08)	358.85	8	< 0.01	97.8
**Memory problems**					
≥ 12 weeks after infection	3.02 (1.15, 7.94)	81.45	4	< 0.01	95.1
Included children-only study	2.69 (1.41, 5.15)	397.96	7	< 0.01	98.2
Pre-vaccination studies only	4.84 (3.28, 7.15)	5.29	3	0.15	43.3
Peer-reviewed publications only	2.70 (0.82, 8.92)	233.63	3	< 0.01	98.7
**Concentration problems**					
Pre-vaccinated studies only	2.39 (0.76, 7.47)	202.11	2	< 0.01	99.0
Peer-reviewed publications only	2.17 (0.77, 6.10)	200.78	2	< 0.01	99.0

df, degrees of freedom.

Heterogeneity between the studies in all analyses was high, with an I^2^ value ranging from to 97.1% to 98.8% in the five main analyses. This reflects the highly variable study designs included in the analysis due to the disparate nature of the available data or the heterogeneity of COVID-19 disease and long COVID itself.

Leave-1-out analyses found the main analyses did not lose statistical significance when each study was individually removed, with the exception of the removal of Carazo 2022^37^ from the concentration analysis, indicating that the results were generally robust.

For all analyses, no evidence of publication bias was found when using the Egger’s test (fatigue *P*-value = 0.53; shortness of breath *P*-value = 0.99; cognition *P*-value = 0.58; memory *P*-value = 0.57; concentration *P*-value = 0.87), and funnel plots generally showed symmetry, indicating publication bias is unlikely.

### Health-related quality of life after SARS-CoV-2 infection

Health-related quality of life was reported in 11 studies (Supplementary Table S8) by validated instruments including the RAND-36, EuroQol-5D (EQ-5D), and 36-Item Short Form Survey (SF-36), and three paediatric instruments: Health Behaviour in School-aged Children (HBSC), Paediatric Quality of Life Inventory (PedsQL), and Children’s Somatic Symptoms Inventory-24 (CSSI-24).

Hospitalised COVID-19 patients’ quality of life was significantly lower in most or all instrument domains when compared to healthy, non-hospitalised uninfected controls^40–42^ but similar to controls hospitalised for pneumonia or other non-COVID-19 reasons^39, 43^. Quality of life comparisons for all SARS-CoV-2 infections were overall inconclusive; two studies reported no differences between cases and controls for adults using the EQ-5D^36^, while two studies found that more COVID-19-positive adults self-reported poorer health compared to the previous year at 3 and 8 months post-infection compared to non-infected controls^44, 45^. In children and/or adolescents, one study reported no significant difference between groups^47^, one found quality of life and sleep significantly better in the SARS-CoV-2-infected adolescents^23^, and one study reported that younger teenagers were more likely to report physical concerns while older teenagers more likely to report mental concerns^46^.

### Methodological quality

Study quality assessed by the modified Newcastle–Ottawa scale found that 21 studies were of low risk of bias/high quality and 12 of medium risk of bias/medium quality. Scores for each study are presented in Supplementary Table S9.

## Discussion

Our systematic review and meta-analyses found that COVID-19 infection results in a significantly raised risk of fatigue (1.72-fold) and shortness of breath (2.60-fold) at four or more weeks post-onset of infection when compared to an uninfected control group. There was also an increased risk of neurological symptoms found in this post-infection period; memory problems had a 1.44-fold increased risk, and concentration problems a 2.53-fold increased risk. Although analyses of only hospitalised or outpatient populations showed a similar risk of fatigue (RR 1.59) or shortness of breath (RR 2.78) to the analyses of all COVID-19 infections, those studies included in the SLR that reported risks from different COVID-19 populations consistently found a higher relative risk of symptoms in intensive care unit (ICU) patients compared to all hospitalised patients^15^. A higher relative risk was also observed in patients with severe infection compared to non-severe infection, and in and non-hospitalised subgroups compared to all COVID-19 infections (adjusted for prior healthcare use and comorbidities)^15, 16, 38^. These studies indicate that severity of infection may have a significant impact on the likelihood of developing long COVID.

While previous systematic reviews of long COVID have aimed to characterise its symptoms, prevalence, and risk factors, reviews and quantitative analyses of risks of common symptoms after infection reported studies with uninfected controls are still limited. A clear understanding of post-COVID symptom risk and prevalence is important for informing healthcare providers and healthcare systems to improve access to resources and investigate new therapeutic strategies. However, many long COVID symptoms are commonly found in the general population and can be caused by other illnesses and infections, while it is also possible that some symptoms, such as fatigue and headache, may have been worsened by other stresses or disruptions associated with the pandemic^48^. This review therefore confirms that SARS-CoV-2 infection, including non-severe infection, is associated with substantially increased risks of developing mid- and long-term symptoms after adjustments for factors such as demographic characteristics and comorbidities.

Some definitions of long COVID require infection to have occurred at least 12 weeks before symptoms. As we included all studies with a minimum follow-up of 4 weeks to enable us to capture a larger number of studies to perform meta-analyses for as many symptoms as possible, we also performed sensitivity analyses that included only those studies with outcomes reported 12 weeks after infection. Relative risks for these were similar to that of the main analyses for fatigue and shortness of breath; while the risk of memory problems was higher than the main analysis (RR 3.02 vs. RR 2.53), this increase was not significantly different and only three studies could be included in the sensitivity analysis.

Sensitivity analyses confirmed that the main analyses of risk of fatigue, shortness of breath, and cognition outcomes were robust, as only two sensitivity analyses resulted in a loss of statistical significance, and these analyses only included a small number of studies. Interestingly, sensitivity analyses including only studies estimated to include a pre-vaccination population found a higher risk ratio than the main analyses for fatigue, shortness of breath, and memory problems which may indicate that vaccination lowers risk of long COVID; however, this increase was not significant.

Health-related quality of life of people after COVID-19 infection compared to uninfected controls was also significantly lower in many or all domains when compared to non-hospitalised controls, but not compared to controls hospitalised for other non-COVID causes, and QoL differences between all COVID-19 infections and their controls were varied, with no statistical analysis of QoL outcomes performed in some studies. However, there is evidence from a number of studies that people with long COVID symptoms report an overall deterioration in quality of life. A recent meta-analysis reported the pooled prevalence of poor quality of life to be 59%^49^. In addition to having a decreased quality of life, patients with long COVID are also likely to report reduced mobility (36%), self-care (8%), and usual activities (28%), increased pain/discomfort (42%), and deteriorated psychological health (38%)^49–51^.

There is evidence that increasing age, female sex, socioeconomic deprivation, smoking, obesity, asthma, and poor pre-pandemic physical and mental health are some of the major risk factors for development of long COVID comorbidities^9, 52^, although we were unable to assess the impact of these on relative risk of long COVID symptoms due to a lack of comparable subpopulations between the included studies. While long COVID can develop regardless of the severity of the initial infection, the severity of the initial COVID-19 infection^53^, as well as the number of symptoms during acute illness, have been indicated as risk factors for long COVID^54^. Studies included in our review that stratified symptom risks by severity support this association, as subgroups of hospitalised patients had a higher risk of fatigue, shortness of breath, and neurocognitive decline than all COVID-19 patients, with an even higher risk for ICU patients^15^. Patients hospitalised with severe COVID-19 illness had a greater risk of cognitive impairment than those hospitalised with non-severe illness^38^.

There is increasing evidence that vaccination against SARS-CoV-2 may decrease the risk of long COVID development after infection. Two studies included in our review reported significantly lowered risks of fatigue and concentration in vaccinated infected cases compared to unvaccinated infected controls^15, 24^. Moreover, a recent systematic review identified 17 studies investigating the impact of COVID-19 vaccines before infection on risk or odds of long COVID, or on changes to long COVID symptoms. The six studies investigating the impact of vaccines before infection found that vaccination is associated with reduced risks, with preliminary evidence indicating that two vaccine doses are more effective than a single dose. Of the 11 studies investigating changes in long COVID symptoms after vaccination, seven studies showed improvements in symptoms at least one dose post-vaccination, while four studies reported either no change or worsening of symptoms^55^.

The variant of SARS-CoV-2 may also influence risk of long COVID. A case–control observational study of vaccinated participants using self-reported data from the UK COVID Symptom Study app found a significant reduction in risk of long COVID at least 4 weeks after infection with the Omicron variant compared to the Delta variant; adjusted odds ratios ranged from 0.24 to 0.50, depending on time since vaccination^56^. Data from the UK’s Office for National Statistics as of 27 May 2022 also show the unadjusted prevalence in triple-vaccinated adults of self-reported long COVID 12 to 16 weeks after infection was 4.5%, 4.2%, and 5.0% for infections compatible with Omicron BA.1, Omicron BA.2, or Delta variants, respectively, although there was no statistical evidence of differences between the three variants. Among double-vaccinated adults, the odds of reporting long COVID of any severity was 48.2% lower for the Omicron variant than Delta variant^57^.

Altogether, the immunity that has been globally built up following multiple infections, vaccinations and boosters, and evolving COVID-19 variants may mean that the risk of long COVID not only varies by country and continent, but is constantly changing as reinfections occur, new variants emerge and improved vaccines are introduced.

This study has a number of limitations. Firstly, it only focuses on a few of the most frequently occurring long COVID symptoms, while a wide range of symptoms associated with long COVID have been reported, including headache, chest pain, loss of smell, dizziness, depression and anxiety, sleep disruption, gastrointestinal issues, and joint, muscle and back pain^48, 58^. Secondly, a large amount of heterogeneity in study design, population and outcomes between studies was present. The different study designs of the studies in this review included retrospective, prospective and ambidirectional cohort studies, case–control studies and cross-sectional studies. Additionally, methods of comparing the infected population with the non-infected controls varied across studies. While we stratified patient populations into those that included all infected people and those from hospitals, some studies with hospital patients did not make it clear whether their population was exclusively people hospitalised for COVID-19 illness or for any reason. Two studies included records from hospital testing laboratories, and these were considered not representative of hospitalised patients or outpatients as they may have included regular testing for healthcare workers or visitors^24, 26^.

While some of the included studies reported outcomes for various subgroups, including age, sex, and infection severity, it was not possible to perform informative subgroup analyses due to lack of comparability and the low number of included studies reporting comparable subgroup data. COVID-19 variants at the time of the study were also poorly reported, and therefore a comparison of risks from different variants was not possible. Multiple time points and different outcome descriptions (particularly for cognition outcomes) were also reported by some studies, and although a feasibility assessment was performed in order to select the most comparable outcomes, this process still carries the risk of subjectivity. A need for standardised and validated COVID-19 research tools to improve the quality and reduce reporting variability has been highlighted by a previous review of long COVID^59^, and standardised symptom outcome descriptions may greatly improve future comparisons between studies. Finally, meta-analyses to determine the pooled reported impact of SARS-CoV-2 on quality of life could not be performed, due to the different HRQoL instruments used by the studies.

This review comprehensively summaries the evidence that COVID-19 infection is followed by a significant risk of fatigue, shortness of breath, and cognitive dysfunction, in both hospitalised and non-hospitalised populations, and hospitalised COVID-19 infections are associated with a lower quality of life in the months after illness. Further research is still necessary to clearly understand the impact of vaccination and acquired immunity on the development of long COVID, as well as the risk of long COVID associated with newer COVID-19 variants. However, the widespread extent of COVID-19 infection and reinfection in the global population will make future studies with confirmed uninfected controls difficult to perform.

Now that the wide breadth of COVID-19 symptoms is gradually becoming clearer, further research into their long-term prevalence is required. While our review identified some studies that reported risk of symptoms at multiple time points, the number of studies were sparse and the overall findings from these were inconclusive. Large cohort studies are necessary to fully understand the potential long-term effects of infection and persistence of symptoms in the years after infection, while the need for clinical trials to address the hypothesized underlying mechanisms of long COVID symptoms has also been acknowledged^60^.

## Methods

### Search strategy

This study was conducted in accordance with the Meta-analysis Of Observational Studies in Epidemiology^61^ and the Preferred Reporting Items for Systematic Reviews and Meta-Analyses (PRISMA) guidelines for conducting and reporting systematic reviews^62^. The study protocol was published via PROSPERO: International Prospective Register of Systematic Reviews (#CRD42022331682) and is available at the following link: https://www.crd.york.ac.uk/prospero/display_record.php?ID=CRD42022331682.

MEDLINE (Ovid), MEDLINE, In-Process Citations, Daily Update & Epubs Ahead-of-Print (Ovid), EMBASE (Ovid), PubMed, National Library of Medicine LitCovid, WHO COVID-19, arXiv (https://arxiv.org/) and Europe PMC (https://europepmc.org/) were searched from January 2020 to March 2022, with no language restrictions. Searches were not limited by publication status (unpublished, published, in press, and in progress). The detailed search strategy is available in Supplementary Table S1. Hand-searching, including searching reference lists of relevant articles, was also performed.

### Eligibility criteria, screening and abstraction

We included full publications meeting the following criteria: (1) non-interventional studies with a control group (including retrospective, prospective, and ambidirectional cohort studies, case–control studies, and cross-sectional studies; (2) conducted in people with SARS-CoV-2 infection diagnosed by testing or clinician-suspected; (3) uninfected controls (including those with COVID-19 symptoms but testing negative, and those with other respiratory infections); (4) reporting the relative risk of fatigue, shortness of breath, or cognitive dysfunction or health-related quality of life measured using validated questionnaires in the case populations compared to control populations; (5) reporting outcomes 4 weeks or more after SARS-CoV-2 infection (6) published from 2020 to the date of the search; and (7) full text in English.

Excluded publication types were editorials, letters, case reports or conference abstracts/proceedings. There was no limit on countries included.

Two reviewers (ZM and MG) independently performed two-stage screening (title/abstract and full text screening), with disagreement resolved by discussion. Data extraction and risk of bias assessment were performed by one reviewer, and a second independent reviewer conducted data checking. Data on study characteristics and the outcomes of interest were extracted.

Data were collected using a standardised data extraction form. Extracted data included the name of the geographical location of the study, data source or study setting, SARS-CoV-2 infection period, population characteristics (including size, age, sex, disease severity, and vaccination status), SARS-CoV-2 test, time between infection and outcomes, and symptom (fatigue, shortness of breath, cognitive dysfunction) risk or QoL outcomes. Risk outcomes extracted included hazard ratio, rate ratio, risk ratio, odds ratio, incidence rate ratio, standardised incidence rate, absolute risk increase, or risk difference measures with 95% confidence intervals (CIs), and the risk description used and adjustments performed. QoL outcomes extracted included the instrument and domains used, and QoL scores or descriptions.

Where the SARS-CoV-2 variant or variants in the study were not reported, circulating variants at the time of the studies’ SARS-CoV-2 infection period were estimated by reviewers using global and national COVID-19 variant surveillance data sources for the included countries.

### Quality assessment

Risk of bias was assessed by a modified Newcastle–Ottawa scale, which assessed study quality in three domains: (1) selection of the study groups; (2) comparability of cohorts on the basis of the design or analysis; and (3) ascertainment of outcomes of interest (Supplementary Table S2). Studies were classified as low risk of bias if they scored 7–9 overall, 4–6 for medium risk of bias, and 0–3 for high risk of bias.

### Statistical analysis

Pairwise meta-analyses were performed for the risk of fatigue, shortness of breath, and cognitive dysfunction outcomes. A feasibility assessment was performed to determine whether it was recommended to combine identified studies in a pairwise meta-analysis, based on study population source, cases and controls, participant characteristics (including age, vaccination status, and disease severity), SARS-CoV-2 infection periods, and outcome reporting. Timepoints reported in the studies were assessed, and the risks from the most comparable timepoints across studies included in the analysis. Where multiple outcome descriptions were reported, the broadest description was used in the analysis. Odds ratios, hazard ratios, rate ratios, and prevalence ratios were considered equal estimates^63^. All are referred to in this paper as ‘risk ratios’ (RR), and the most adjusted RR were log transformed and used in analysis. Absolute risk increases and risk differences were not included in any analyses. Weights were calculated using the inverse variance method (weight = 1/variance). Random-effects DerSimonian and Laird models^64^ were fitted to calculate pooled RR and 95% CI for all outcomes.

Heterogeneity was measured using the Cochran’s Q statistic with statistical significance set at *P* < 0.10 (due to a limitation of the Q test that it may be underpowered when the number of studies is low, a higher *P*-value threshold for statistical significance is recommended in these situations) and quantified by the I^2^ test. Publication bias was assessed with funnel plots and the Egger’s test^65^.

The robustness of the results was evaluated using the leave-1-out method^66^ to assess the effect on pooled estimates of removing individual studies. Subgroup analyses of hospitalised or outpatient studies were also performed where possible. A range of sensitivity analyses were conducted where possible, and included (1) only studies that had a minimum of 12 weeks between infection and the reporting of symptoms; (2) addition of a single study removed for potential population overlap; (3) inclusion of children-only studies; (4) only studies where SARS-CoV-2 infection occurred before introduction of COVID-19 vaccination; (5) removal of non-adjusted risks; and (6) exclusion of pre-print studies. Sensitivity analyses were performed if three or more studies could be included in the analysis.

All statistical analyses were conducted using R version 3.5.1, using the packages metafor and forestplot.

### Supplementary Information

Supplementary Information.

## Data Availability

All data analysed in this work were extracted from published materials. Additional extracted data can be obtained upon reasonable request from the corresponding author, Z.M.
