# Supplementary material for: Risk of long COVID main symptoms after SARS-CoV-2 infection: a systematic review and meta-analysis
Source: Sci Rep. 2023 Sep 15;13:15332. doi: 10.1038/s41598-023-42321-9 (PMC10504382; doi:10.1038/s41598-023-42321-9)
Supplement: Supplementary file 1 — Supplementary Information. [file 41598_2023_42321_MOESM1_ESM.docx]

**Supplementary Material**

## Figure S1: Risk of fatigue in hospitalised/outpatient COVID-19 patients (random effects)


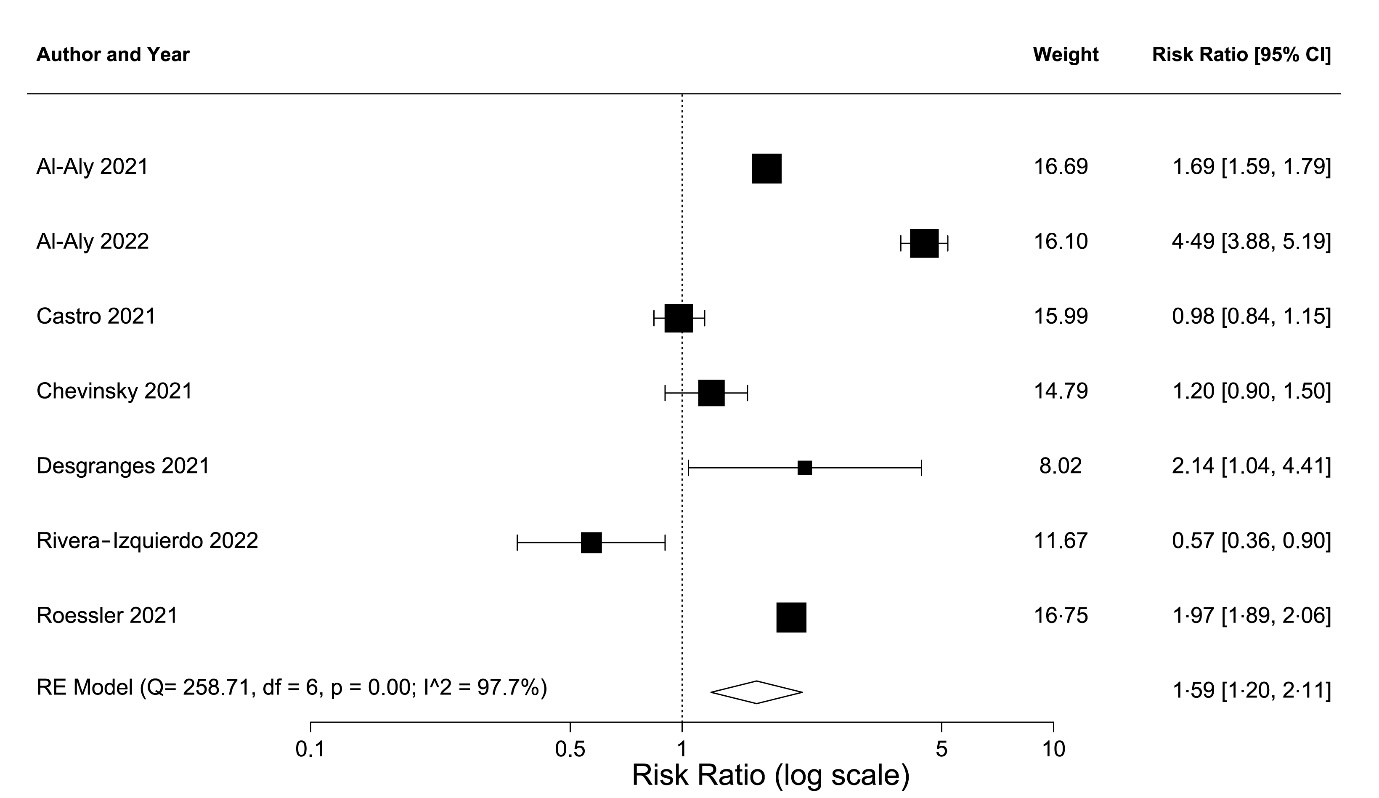
df, degrees of freedom; RE, random effects

## Figure S2: Risk of shortness of breath in hospitalised/outpatient COVID-19 patients (random effects)


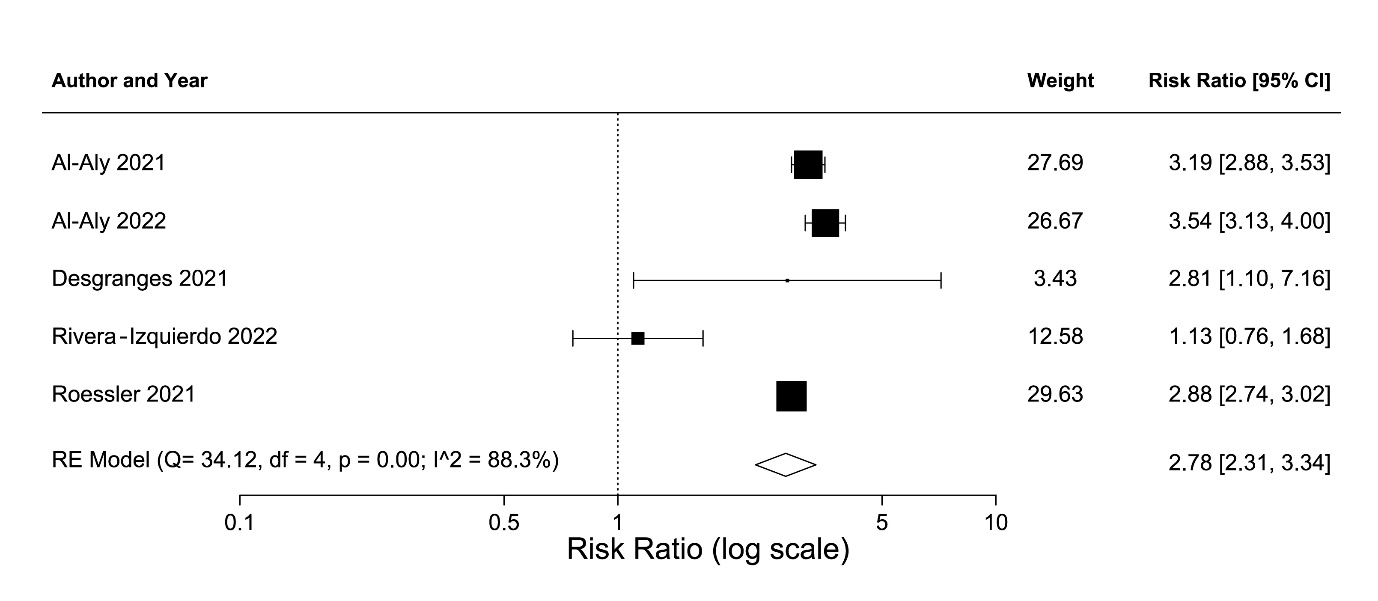
 df, degrees of freedom; RE, random effects

## Table S1: Search strategy

**Embase (Ovid): 2020-2022/03/21**

**Searched 22.3.22**

| 1 | (PASC or "chronic covid syndrome$").ti,ab,ot,kf,kw | 379 |
| --- | --- | --- |
| 2 | ("post acute" adj2 sequela$ adj2 (covid or coronavirus or coronovirus or "corona virus" or COV)).ti,ab,ot,kw. | 73 |
| 3 | (("long$ term$" or longterm$ or "long$ haul$" or longhaul$ or "long$ tail$" or longtail$ or longduration$ or "long duration$" or longlast$ or "long last$" or longstanding$ or "long standing$" or "medium$ term$" or mediumterm$ or "late effect$" or recurren$ or prolong$ or post-viral$ or chronic$ or postacute or "post acute" or persistent$) adj3 (covid$ or coronavirus$ or corona$ virus$ or coronovirus$ or corono$ virus$ or coronavirinae$ or corona$ virinae$ or Cov or "2019-nCoV$" or 2019nCoV$ or "19-nCoV$" or 19nCoV$ or nCoV2019$ or "nCoV-2019$" or nCoV19$ or "nCoV-19$" or "HCoV-19$" or HCoV19$ or "HCoV-2019$" or HCoV2019$ or "2019 novel$" or Ncov$ or "n-cov" or "SARS-CoV-2$" or "SARSCoV-2$" or "SARSCoV2$" or "SARS-CoV2$" or SARSCov19$ or "SARS-Cov19$" or "SARSCov-19$" or "SARS-Cov-19$" or SARSCov2019$ or "SARS-Cov2019$" or "SARSCov-2019$" or "SARS-Cov-2019$" or SARS2$ or "SARS-2$" or SARScoronavirus2$ or "SARS-coronavirus-2$" or "SARScoronavirus 2$" or "SARS coronavirus2$" or SARScoronovirus2$ or "SARS-coronovirus-2$" or "SARScoronovirus 2$" or "SARS coronovirus2$" or "severe acute respiratory syndrome$")).ti,ab,ot,kw. | 4474 |
| 4 | (longcovid$ or long covid$ or longcoronavirus$ or longcorona$ virus$ or long coronavirus$ or long corona$ virus$ or longcoronovirus$ or longcorono$ virus$ or long coronovirus$ or long corono$ virus$ or longcoronavirinae$ or longcorona$ virinae$ or long coronavirinae$ or long corona$ virinae$ or longCov or long Cov or longsars$ or long sars$ or "long severe acute respiratory syndrome$" or longncov$ or long ncov$ or longhcov$ or long hcov$ or "post-acute covid" or "post-acute corona$" or "post-acute corono$" or "post-acute cov" or "post-acute ncov" or "post-acute sars" or "post-acute severe respiratory syndrome$").ti,ab,ot,kw. | 1552 |
| 5 | ((long$ or endur$ or legacy$ or slow$ or gradual$ or protract$ or lengthy$ or chronic$ or persist$ or relaps$ or remit$ or remission$ or residual$ or delay$ or prolong$ or extend$ or linger$ or permanent$ or fluctuat$ or sequela$ or multisystem$ or "multi system$" or nonrecover$ or "non recover$" or subacute$ or "sub acute$" or lasting$ or continuous$ or continual$ or continuing$ or postacute$ or "post acute$" or postdischarg$ or "post discharg$" or postinfect$ or "post infect$" or postviral$ or "post viral$" or postvirus$ or "post virus$" or "late effect$") adj2 (covid$ or coronavirus$ or corona$ virus$ or coronovirus$ or corono$ virus$ or coronavirinae$ or corona$ virinae$ or Cov or "2019-nCoV$" or 2019nCoV$ or "19-nCoV$" or 19nCoV$ or nCoV2019$ or "nCoV-2019$" or nCoV19$ or "nCoV-19$" or "HCoV-19$" or HCoV19$ or "HCoV-2019$" or HCoV2019$ or "2019 novel$" or Ncov$ or "n-cov" or "SARS-CoV-2$" or "SARSCoV-2$" or "SARSCoV2$" or "SARS-CoV2$" or SARSCov19$ or "SARS-Cov19$" or "SARSCov-19$" or "SARS-Cov-19$" or SARSCov2019$ or "SARS-Cov2019$" or "SARSCov-2019$" or "SARS-Cov-2019$" or SARS2$ or "SARS-2$" or SARScoronavirus2$ or "SARS-coronavirus-2$" or "SARScoronavirus 2$" or "SARS coronavirus2$" or SARScoronovirus2$ or "SARS-coronovirus-2$" or "SARScoronovirus 2$" or "SARS coronovirus2$" or "severe acute respiratory syndrome$")).ti,ab,ot,kw. | 6496 |
| 6 | (("long$ term$" or longterm$ or "long$ haul$" or longhaul$ or "long$ tail$" or longtail$ or longduration$ or "long duration$" or longlast$ or "long last$" or longstanding$ or "long standing$" or "medium$ term$" or mediumterm$ or "post-virus$" or "post-viral") adj3 (covid$ or coronavirus$ or corona$ virus$ or coronovirus$ or corono$ virus$ or coronavirinae$ or corona$ virinae$ or Cov or "2019-nCoV$" or 2019nCoV$ or "19-nCoV$" or 19nCoV$ or nCoV2019$ or "nCoV-2019$" or nCoV19$ or "nCoV-19$" or "HCoV-19$" or HCoV19$ or "HCoV-2019$" or HCoV2019$ or "2019 novel$" or Ncov$ or "n-cov" or "SARS-CoV-2$" or "SARSCoV-2$" or "SARSCoV2$" or "SARS-CoV2$" or SARSCov19$ or "SARS-Cov19$" or "SARSCov-19$" or "SARS-Cov-19$" or SARSCov2019$ or "SARS-Cov2019$" or "SARSCov-2019$" or "SARS-Cov-2019$" or SARS2$ or "SARS-2$" or SARScoronavirus2$ or "SARS-coronavirus-2$" or "SARScoronavirus 2$" or "SARS coronavirus2$" or SARScoronovirus2$ or "SARS-coronovirus-2$" or "SARScoronovirus 2$" or "SARS coronovirus2$" or "severe acute respiratory syndrome$")).ti,ab,ot,kw. | 1595 |
| 7 | ((postcovid$ or post covid$ or postcoronavirus$ or postcorona$ virus$ or post coronavirus$ or post corona$ virus$ or postcoronovirus$ or postcorono$ virus$ or post coronovirus$ or post corono$ virus$ or postcoronavirinae$ or postcorona$ virinae$ or post coronavirinae$ or post corona$ virinae$ or postCov or post Cov or postsars$ or post sars$ or "post severe acute respiratory syndrome$" or postncov$ or post ncov$ or posthcov$ or post hcov$) adj3 (syndrome$ or disorder$ or illness$ or sickness$ or disease$ or condition$ or symptom$ or sign$ or prognos$ or followup$ or "follow up$" or feature$ or comorbid$ or "co morbid$" or multimorbid$ or "multi morbid$" or survivor$ or survival$ or risk$ or care$ or convalescen$ or recuperat$ or aftercare$ or ambulatory$ or outpatient$ or "out patient$")).ti,ab,ot,kw. | 1045 |
| 8 | ((ongoing$ or long$ or endur$ or legacy$ or slow$ or gradual$ or protract$ or lengthy$ or chronic$ or persist$ or relaps$ or remit$ or remission$ or residual$ or delay$ or prolong$ or extend$ or linger$ or permanent$ or fluctuat$ or multisystem$ or "multi system$" or nonrecover$ or "non recover$" or subacute$ or "sub acute$" or lasting$ or continuous$ or continual$ or continuing$ or postacute$ or "post acute$" or postdischarg$ or "post discharg$" or postinfect$ or "post infect$" or postviral$ or "post viral$" or postvirus$ or "post virus$" or "medium$ term$" or mediumterm$) adj4 (sequela$ or illness$ or symptom$ or sign$ or prognos$ or rehab$ or convalescen$ or recuperat$ or followup$ or "follow up$" or feature$) adj10 (covid$ or coronavirus$ or corona$ virus$ or coronovirus$ or corono$ virus$ or coronavirinae$ or corona$ virinae$ or Cov or "2019-nCoV$" or 2019nCoV$ or "19- nCoV$" or 19nCoV$ or nCoV2019$ or "nCoV-2019$" or nCoV19$ or "nCoV-19$" or "HCoV-19$" or HCoV19$ or "HCoV-2019$" or HCoV2019$ or "2019 novel$" or Ncov$ or "n-cov" or "SARS-CoV-2$" or "SARSCoV-2$" or "SARSCoV2$" or "SARS-CoV2$" or SARSCov19$ or "SARS-Cov19$" or "SARSCov-19$" or "SARS-Cov-19$" or SARSCov2019$ or "SARS-Cov2019$" or "SARSCov-2019$" or "SARS-Cov-2019$" or SARS2$ or "SARS-2$" or SARScoronavirus2$ or "SARS-coronavirus-2$" or "SARScoronavirus 2$" or "SARS coronavirus2$" or SARScoronovirus2$ or "SARS-coronovirus-2$" or "SARScoronovirus 2$" or "SARS coronovirus2$" or "severe acute respiratory syndrome$")).ti,ab,ot,kw. | 3109 |
| 9 | or/1-8 | 10769 |
| 10 | ((chronic$ or Long or post-acute or longterm or late or persistent$) adj3 (sequela$ or effect$ or symptom$)).ti,ab,ot,kf,kw. | 271817 |
| 11 | incidence/ | 493298 |
| 12 | hazard ratio/ | 61518 |
| 13 | morbidity/ | 376963 |
| 14 | disease activity/ | 84084 |
| 15 | disease progression/ | 121675 |
| 16 | incidence density rate/ | 47 |
| 17 | Prevalence/ | 835865 |
| 18 | standardized incidence ratio/ | 3426 |
| 19 | exp cumulative incidence/ | 5103 |
| 20 | (morbid$ or epidemiolog$ or demograph$).ti,ab,ot,kw,kf. | 1848227 |
| 21 | (occurrence$ or incidence$ or prevalence$ or episode$).ti,ab,ot,kw,kf. | 2846313 |
| 22 | (relative risk or hazard ratio or rate ratio or risk ratio or odds ratio).ti,ab,ot,kw,kf. | 602015 |
| 23 | or/10-22 | 5206633 |
| 24 | Productivity/ | 44467 |
| 25 | Absenteeism/ | 18651 |
| 26 | Caregiver Burden/ | 9272 |
| 27 | Caregiver/ | 97458 |
| 28 | Work Disability/ | 5492 |
| 29 | ((human$ or social$ or societ$ or work$ or employe$ or business$ or communit$ or famil$ or carer$ or caregiver$) adj3 (burden$ or consequenc$ or impact$ or problem$ or productivity or sickness or impairment$)).ti,ab,ot,hw. | 198689 |
| 30 | ((long standing or longstanding or long term or longterm or permanent or employee$) adj2 (absence$ or absent$ or ill$ or sick$ or disab$)).ti,ab,ot,hw. | 16837 |
| 31 | (llsi or ((emergenc$ or domestic$ or famil$ or carer$ or caregiver$) adj3 leave$)).ti,ab,ot. | 1277 |
| 32 | (burden adj2 (illness$ or disease$ or sickness$)).ti,ab,ot,hw. | 73106 |
| 33 | ((allowance or status or long-term or pension$ or benefit$) adj2 (disab$ or incapacit$)).ti,ab,ot,hw. | 32846 |
| 34 | ((unable or inability or incapacit$ or incapab$) adj3 work).ti,ab,ot,hw. | 2981 |
| 35 | ((health or healthcare) adj2 (resource$ or fund$)).ti,ab,ot,hw. | 52113 |
| 36 | ("length of stay" or "duration of stay" or "extended stay" or "prolonged stay").ti,ab,ot,hw. | 250750 |
| 37 | ((ambulatory or ambulance or hospital or A&E or emergency) adj2 (attention$ or trip or trips or visit$ or stay$ or admission$ or admitted or transport$)).ti,ab,ot,hw. | 484393 |
| 38 | ((clinic or clinics or outpatient$ or out-patient$ or surger$) adj2 (appointment$ or attention$ or trip or trips or visit$)).ti,ab,ot,kw,kf. | 52779 |
| 39 | ((hospital$ or inpatient$ or in-patient$) adj2 (stay$ or admission$ or episode$)).ti,ab,ot,kw,kf. | 278505 |
| 40 | ((length$ or hospital$ or inpatient$ or in-patient$) adj2 stay$).ti,ab,ot,kw,kf. | 288110 |
| 41 | ((resource$ or healthcare or service$) adj3 "use").ti,ab,ot,kw,kf. | 66338 |
| 42 | ((outpatient$ or outpatient$ or inpatient$ or in-patient$ or ambulatory or emergenc$) adj2 care).ti,ab,ot,kw,kf. | 93408 |
| 43 | ((GP or general practitioner$ or doctor$ or inpatient$ or in-patient$ or clinician$ or specialist$ or physician$ or clinic or clinics or outpatient$ or out-patient$ or surger$) adj2 (appointment$ or attention$ or trip or trips or visit$)).ti,ab,ot,kw,kf. | 77981 |
| 44 | ((cost or costs or burden) adj2 (illness$ or disease$ or sickness$)).ti,ab,ot,kw,kf. | 59302 |
| 45 | (cost$ adj2 (burden$ or estimat$ or variabl$ or hospital$ or control$)).ti,ab,ot,kw,kf. | 74851 |
| 46 | ((resource$ or healthcare or service$) adj3 (uses or usage or utilis$ or utiliz$ or consum$ or consuming or consumption$)).ti,ab,ot,kw,kf. | 85006 |
| 47 | ((hospitali?ation? or hospital admission? or patient admission?) and ("length of stay?" or "length of hospital stay$" or "duration of stay$" or "duration of hospital stay$" or cost?)).ti,ab,ot,kf,kw. | 79705 |
| 48 | (Health adj2 (expenditure$ or expense$ or care cost$)).ti,ab,ot,kf,kw. | 36640 |
| 49 | (utili?ation or allocat$).ti,ab,ot,kf,kw. | 505527 |
| 50 | (economic impact or economic cost$).ti,ab,ot,kf,kw. | 25204 |
| 51 | (social impact or societal impact or social cost$ or societal cost$).ti,ab,ot,kf,kw. | 10582 |
| 52 | ((disease or illness or disabilit$ or severity or trajectory or progress$) and (cost? or economic?)).ti,ab,ot,kf,kw. | 281466 |
| 53 | (direct payment? or direct service payment?).ti,ab,ot,kf,kw. | 289 |
| 54 | ((health or healthcare or health care or direct service or indirect or in-direct or hospital or drug$) adj2 cost$).ti,ab,ot,kf,kw. | 111787 |
| 55 | ((human$ or famil$ or carer$ or caregiver$) adj3 (burden$ or consequenc$ or impact$ or problem$ or productivity or sickness or impairment$)).ti,ab,ot,kw,kf. | 66204 |
| 56 | (reimburs$ or payer$1).ti,ab,ot,kw,kf. | 74439 |
| 57 | health care cost$.ti,ab,ot,kw,kf. | 24719 |
| 58 | (cost utility analysis or cost control$ or economic aspect).ti,ab,ot,kw,kf. | 7409 |
| 59 | (economic$ adj2 (burden$ or consequenc$ or impact$ or problem$)).ti,ab,ot,kw,kf. | 53038 |
| 60 | ((health or healthcare) adj2 (resource$ or fund$)).ti,ab,ot,kw,kf. | 53376 |
| 61 | (unit cost or unit costs).ti,ab,ot,kw,kf. | 5060 |
| 62 | (cost adj2 (estimat$ or variable$ or mini$)).ti,ab,ot,kw,kf. | 28409 |
| 63 | (fiscal or financial or finance or funding).ti,ab,ot,kw,kf. | 250317 |
| 64 | (financ$ manag$ or health care cost$ or health care financ$ or health economic$ or cost minimization analysi$).ti,ab,ot,kw,kf. | 47042 |
| 65 | (socioeconomic$ or socio-economic$ or cost benefit analys$ or cost effecti$ analys$ or economic$ evaluat$ or cost utility analysis or cost control$ or economic aspect).ti,ab,ot,kw,kf. | 233705 |
| 66 | "cost benefit analysis"/ | 89831 |
| 67 | "cost of illness"/ | 20508 |
| 68 | "cost control"/ | 72576 |
| 69 | economic aspect/ or financial management/ | 230523 |
| 70 | exp "health care cost"/ | 315754 |
| 71 | health economics/ | 34107 |
| 72 | health care utilization/ or "procedures and techniques utilization"/ | 83052 |
| 73 | socioeconomics/ or exp economic status/ | 284470 |
| 74 | or/24-73 | 2929455 |
| 75 | exp quality of life/ | 572475 |
| 76 | (sf36 or sf 36 or sf-36 or short form 36 or shortform 36 or sf thirtysix or sf thirty six or shortform thirtysix or shortform thirty six or short form thirty six or short form thirtysix or short form thirty six).ti,ab,ot. | 45993 |
| 77 | (sf6 or sf 6 or sf-6 or short form 6 or shortform 6 or sf six or sfsix or shortform six or short form six).ti,ab,ot. | 2693 |
| 78 | (sf12 or sf 12 or sf-12 or short form 12 or shortform 12 or sf twelve or sftwelve or shortform twelve or short form twelve).ti,ab,ot. | 10902 |
| 79 | (sf6D or sf 6D or sf-6D or short form 6D or shortform 6D or sf six D or sfsixD or shortform six D or short form six D).ti,ab,ot. | 1689 |
| 80 | (sf20 or sf 20 or sf-20 or short form 20 or shortform 20 or sf twenty or sftwenty or shortform twenty or short form twenty).ti,ab,ot. | 487 |
| 81 | (sf8 or sf 8 or sf-8 or short form 8 or shortform 8 or sf eight or sfeight or shortform eight or short form eight).ti,ab,ot. | 1101 |
| 82 | "health related quality of life".ti,ab,ot. | 74601 |
| 83 | (Quality adjusted life or Quality-adjusted-life).ti,ab,ot. | 23228 |
| 84 | "assessment of quality of life".ti,ab,ot. | 3248 |
| 85 | (eq-5d or eq5d or eq-5 or eq5 or euro qual or euroqual or euro qual5d or euroqual5d or euro qol or euroqol or euro qol5d or euroqol5d or euro quol or euroquol or euro quol5d or euroquol5d or eur qol or eurqol or eur qol5d or eur qol5d or eur?qul or eur?qul5d or euro$ quality of life or european qol).ti,ab,kf. | 26500 |
| 86 | (euro$ adj3 (5 d or 5d or 5 dimension$ or 5dimension$ or 5 domain$ or 5domain$)).ti,ab,kf. | 7717 |
| 87 | (hql or hrql or hqol or h qol or hrqol or hr qol).ti,ab,ot. | 40176 |
| 88 | (hye or hyes).ti,ab,ot. | 149 |
| 89 | health$ year$ equivalent$.ti,ab,ot. | 41 |
| 90 | (hui or hui1 or hui2 or hui3 or hui4 or hui-4 or hui-1 or hui-2 or hui-3).ti,ab,ot. | 3567 |
| 91 | (quality time or qwb or "quality of well being" or "quality of wellbeing" or "index of wellbeing" or "index of well being").ti,ab,ot,hw. | 1389 |
| 92 | (Disability adjusted life or Disability-adjusted life or health adjusted life or health-adjusted life or "years of healthy life" or healthy years equivalent or "years of potential life lost" or "years of health life lost").ti,ab,ot. | 6046 |
| 93 | (QALY$ or DALY$ or HALY$ or YHL or HYES or YPLL or YHLL or qald$ or qale$ or qtime$ or AQoL$).ti,ab,ot. | 29817 |
| 94 | (qald$ or qale$ or qtime$).ti,ab,kf. | 383 |
| 95 | (timetradeoff or time tradeoff or time trade-off or time trade off or TTO or Standard gamble$ or "willingness to pay").ti,ab,ot. | 14501 |
| 96 | 15d.ti,ab,ot. | 2771 |
| 97 | (HSUV$ or health state$ value$ or health state$ preference$ or HSPV$).ti,ab,ot. | 701 |
| 98 | (utilit$ adj3 ("quality of life" or valu$ or scor$ or measur$ or health or life or estimat$ or elicit$ or disease$ or cost$ or disease$ or mean or gain or gains or index$)).ti,ab,ot. | 32818 |
| 99 | (utilities or disutili$).ti,ab,ot. | 14020 |
| 100 | (illness state$1 or health state$1).ti,ab,kf. | 13103 |
| 101 | (multiattribute$ or multi attribute$).ti,ab,kf. | 1338 |
| 102 | Cost-Benefit Analysis/ and (cost-effectiveness ratio$ and (perspective$ or life expectanc$)).ti,ab,kf. | 1026 |
| 103 | ((quality of life or qol) adj (score$1 or measure$1)).ti,ab,kf. | 32723 |
| 104 | (PAC-19QOL or PICQoL or CRQ or BFI or "D-FIS" or MAF or MFI or MFSI or MMSE or HEIQ or WURSS or "4DSQ" or "Basis-32" or BPRS or BSI or "CES-D" or "EWPS$ or GAS or GHQ or LSQ-1 or LSQ1 or LSQ-2 or LSQ2 or PAIS or PAIS-SR or POMS or PRIME-MD or PRIME-MD PHQ or QLQ-IR or QLQ-SR or QPD Panel or RBMY or SCL-90R or SCL90R or SCL-90R or SCL90-R or VABS or W-QLI or WQLI or CDS or CES-D or DUKE-AD or GD" or HADS or MADRS or MDI or QLDS or RDS or SDI or SDSS).ti,ab,ot. | 101989 |
| 105 | ("Paediatric Intensive Care Quality of Life questionnaire" or "Chronic Respiratory Disease Questionnaire" or "Brief Fatigue Inventory" or "Daily Fatigue Impact Scale" or "Dutch Exertion Fatigue Scale" or "Dutch Fatigue Scale" or "Fatigue Symptom Inventory" or "Multidimensional Assessment of Fatigue" or "Multidimensional Fatigue Inventory" or "Modified Fatigue Impact Scale" or "Multidimensional Fatigue Symptom Inventory" or "Mini Mental State Examination" or "Health Education Impact Questionnaire" or "Wisconsin Upper Respiratory Symptom Survey" or "Four-Dimensional Symptom Questionnaire" or "Behavior and Symptom Identification Scale" or "Brief Psychiatric Rating Scale" or "Brief Symptom Inventory" or "Center for Epidemiologic Studies Depression Scale" or "Endicott Work Productivity Scale" or "global Assessment Scale").ti,ab,ot,kf,kw. | 37079 |
| 106 | ("General Health Questionnaire" or "Mayers' LSQ" or "mayers Lifestyle Questionnaire$" or "Psychosocial Adjustment to Illness Scale" or "Profile of Mood States" or "Primary Care Evaluation of Mental Disorders Patient Health Questionnaire" or "Oregon Quality of Life" or "Quick PsychoDiagnostics Panel" or "Quick Psycho-Diagnostics Panel" or "Rivermead Behavioral Memory Test" or "Symptom Checklist-90-Revised" or "Vineland Adaptive Behaviour Scale$" or "Wisconsin Quality of Life Index" or "Carroll Rating Scale for Depression" or "Center for Epidemiologic Studies Depression Scale" or "Duke Anxiety Depression Scale" or "Geriatric Depression Scale" or "Hospital Anxiety and Depression scale").ti,ab,ot,kf,kw. | 38162 |
| 107 | ("Montgomery-Asberg Depression Rating Scale" or "Major Depression Inventory" or "Quality of Life in Depression Scale" or "Rand 8-item Depression Screener" or "Short Depression Interview" or "Signs of Depression Screening Scale").ti,ab,ot,kf,kw. | 4339 |
| 108 | or/75-107 | 779125 |
| 109 | or/23,74,108 | 7658112 |
| 110 | Clinical study/ | 157579 |
| 111 | Case control study/ | 185674 |
| 112 | Family study/ | 25397 |
| 113 | Longitudinal study/ | 169650 |
| 114 | Retrospective study/ | 1218698 |
| 115 | Prospective study/ | 753973 |
| 116 | Randomized controlled trials/ or Random$.tw. or placebo$.mp. or double-blind$.tw. or ("case study" or "case studies" or "case report" or "case reports" or "case series").tw. | 2908296 |
| 117 | 115 not 116 | 616506 |
| 118 | Cohort analysis/ | 820617 |
| 119 | (Cohort adj (study or studies)).mp. | 392492 |
| 120 | (Case control adj (study or studies)).tw. | 152389 |
| 121 | (follow up adj (study or studies)).tw. | 68742 |
| 122 | (observational adj (study or studies)).tw. | 212413 |
| 123 | (epidemiologic$ adj (study or studies)).tw. | 115038 |
| 124 | (cross sectional adj (study or studies)).tw. | 281694 |
| 125 | or/110-114,117-124 | 3257089 |
| 126 | animal/ or animal experiment/ | 4333457 |
| 127 | (rat or rats or mouse or mice or murine or rodent or rodents or hamster or hamsters or pig or pigs or porcine or rabbit or rabbits or animal or animals or dogs or dog or cats or cow or bovine or sheep or ovine or monkey or monkeys).ti,ab,ot,hw. | 7202525 |
| 128 | or/126-127 | 7202525 |
| 129 | exp human/ or human experiment/ | 23400480 |
| 130 | 128 not (128 and 129) | 5458603 |
| 131 | 125 not 130 | 3204430 |
| 132 | 131 not (letter or editorial).pt. | 3156765 |
| 133 | limit 132 to yr="2020 -Current" | 725195 |
| 134 | 9 and 109 and 133 | 2293 |

## Table S2: Modified Newcastle-Ottawa scale Quality assessment

| **Question** | **Decision** | **Stars awarded** | |
| --- | --- | --- | --- |
| **Selection (max 4 stars)** | | |  |
| Is the case definition adequate? | Yes, with independent validation (e.g., >1 person/record/time/process to extract information, or reference to primary record source such laboratory reports or medical/hospital records) | 1 | |
|  | Yes, with record linkage (e.g., ICD codes in database) or self-report with no reference to primary record | 1 | |
|  | No description | 0 | |
| Representativeness of the cases | All eligible cases with outcome of interest over a defined period of time, all cases in a defined catchment area, all cases in a defined hospital or clinic, group of hospitals, health maintenance organisation, or an appropriate sample of those cases (e.g., random sample) | 1 | |
|  | Potential for selection biases or not stated (e.g., convenience sampling) | 0 | |
| Selection of controls | Community controls (i.e., Covid-19 negative, general population, healthy population, outpatients, influenza or other illness patients) | 1 | |
|  | Hospital controls (i.e., inpatients, hospital employees) | 1 | |
|  | No description | 0 | |
| Definition of controls | No history of disease (i.e., laboratory confirmed COVID-19 negative) | 1 | |
|  | No description (e.g., volunteers or untested population) | 0 | |
| **Comparability (max 2 stars)** | | |  |
| Case/control matching/adjustments | Cases and controls are matched for age and sex in the design and/or adjusted for in the analysis | 1 | |
|  | Cases and controls are matched for other confounders (e.g. co-morbidities, race/ethnicity, socio-economic status, education) in the design and/or adjusted for in the analysis. | + 1 | |
| **Outcome (max 3 stars)** | | |  |
| Assessment of outcome | Record linkage (e.g., identified through ICD codes on database records) | 1 | |
|  | Self-report (i.e., no reference to original medical records or laboratory reports to confirm the outcome) | 1 | |
|  | No description | 0 | |
| Was follow-up long enough for outcomes to occur? | Yes (i.e., >4 weeks) | 1 | |
|  | No (i.e., unclear if all patients <4 weeks) | 0 | |
| Adequacy of follow Up of cohorts | Complete follow up for all subjects | 1 | |
|  | Subjects lost to follow up unlikely to introduce bias (i.e., follow up rate > 80%), or description provided of those lost | 1 | |
|  | Follow up rate < 80% and no description of those lost | 0 | |
|  | No statement | 0 | |

## Table S3: Studies excluded at full text screening

| **Study** | **Reason for exclusion** |
| --- | --- |
| Abdelghani, Mohamed, et al. "Sleep disturbances following recovery from COVID-19: a comparative cross-sectional study in Egypt." Eastern Mediterranean Health Journal 28.1 (2022): 14-22. | Wrong outcomes |
| Al-Aly, Ziyad, Yan Xie, and Benjamin Bowe. "Burdens of Post-acute Sequelae of COVID-19 by Age, Race, Sex, and Health Status." (2021). | Wrong outcomes |
| Aranyo, Julia, et al. "B-PO02-167 INAPPROPRIATE SINUS TACHYCARDIA IN POST-COVID-19 SYNDROME." Heart Rhythm 18.8 (2021): S166. | Wrong comparator |
| Ayoubkhani, Daniel, et al. "Post-covid syndrome in individuals admitted to hospital with covid-19: retrospective cohort study." bmj 372 (2021). | Wrong outcomes |
| Blankenburg, Judith, et al. "Comparison of mental health outcomes in seropositive and seronegative adolescents during the COVID19 pandemic." Scientific reports 12.1 (2022): 1-8. | Wrong study duration |
| Boiko, Dmytro I., et al. "Circadian rhythm disorder and anxiety as mental health complications in post-COVID-19." Environmental Science and Pollution Research 29.19 (2022): 28062-28069. | Wrong outcomes |
| Carter, Stephen J., et al. "Functional status, mood state, and physical activity among women with post-acute COVID-19 syndrome." medRxiv (2022). | Wrong outcomes |
| Nzeako, Ihechiluru, Feng Li, and Paul Thuluvath. "Long-term Symptoms After COVID-19 Compared to a Control Group." The American Journal of Gastroenterology (2021). | Wrong outcomes |
| Cohen, Ken, et al. "Risk of persistent and new clinical sequelae among adults aged 65 years and older during the post-acute phase of SARS-CoV-2 infection: retrospective cohort study." bmj 376 (2022). | Wrong study duration |
| Del Brutto, Oscar H., et al. "Cognitive sequelae of long COVID may not be permanent: A prospective study." European Journal of Neurology 29.4 (2022): 1218-1221. | Wrong outcomes |
| Delgado-Alonso, Cristina, et al. "Cognitive dysfunction associated with COVID-19: A comprehensive neuropsychological study." Journal of Psychiatric Research 150 (2022): 40-46. | Wrong outcomes |
| Dennis, Andrea, et al. "Multiorgan impairment in low-risk individuals with post-COVID-19 syndrome: a prospective, community-based study." BMJ open 11.3 (2021): e048391. | Wrong study design |
| Cirulli, Elizabeth T., et al. "Long-term COVID-19 symptoms in a large unselected population." medrxiv (2020). | Wrong outcomes |
| Pauley, Ellen, et al. "Recovery from Covid-19 critical illness: A secondary analysis of the ISARIC4C CCP-UK cohort study and the RECOVER trial." Journal of the Intensive Care Society (2021): 17511437211052226. | Wrong outcomes |
| Erdal, Yuksel, et al. "Autonomic dysfunction in patients with COVID‑19." Acta Neurologica Belgica (2022): 1-7. | Wrong outcomes |
| Ferrando, Stephen J., et al. "Neuropsychological, Medical, and Psychiatric Findings After Recovery from Acute COVID-19: A Cross-sectional Study." Journal of the Academy of Consultation-liaison Psychiatry (2022). | Wrong comparator |
| Fink, Thais T., et al. "Persistent symptoms and decreased health-related quality of life after symptomatic pediatric COVID-19: A prospective study in a Latin American tertiary hospital." Clinics 76 (2021). | Wrong study duration |
| Galván-Tejada, Carlos E., et al. "Persistence of COVID-19 symptoms after recovery in Mexican population." International journal of environmental research and public health 17.24 (2020): 9367. | Wrong study duration |
| Graham, Edith L., et al. "Persistent neurologic symptoms and cognitive dysfunction in non‐hospitalized Covid‐19 “long haulers”." Annals of clinical and translational neurology 8.5 (2021): 1073-1085. | Wrong population |
| Guo, Panyuan, et al. "COVCOG 2: Cognitive and Memory Deficits in Long COVID: A Second Publication From the COVID and Cognition Study." Frontiers in aging neuroscience 14 (2022). | Wrong outcomes |
| Hack, Laura M., et al. "Survivors of SARS-CoV-2 infection show neuropsychiatric sequelae measured by surveys, neurocognitive testing, and magnetic resonance imaging: Preliminary results." medRxiv (2021). | Wrong outcomes |
| Haroon, Shamil, et al. "Protocol: Therapies for Long COVID in non-hospitalised individuals: from symptoms, patient-reported outcomes and immunology to targeted therapies (The TLC Study)." BMJ Open 12.4 (2022). | Protocol |
| Henneghan, Ashley M., et al. "Describing cognitive function and psychosocial outcomes of COVID-19 survivors: A cross-sectional analysis." Journal of the American Association of Nurse Practitioners 34.3 (2022): 499-508. | Wrong comparator |
| Jandhyala, Ravi. "Design, validation and implementation of the post-acute (long) COVID-19 quality of life (PAC-19QoL) instrument." Health and quality of life outcomes 19.1 (2021): 1-11. | Wrong study design |
| Deuel, Jeremy Werner, et al. "Persistence, prevalence, and polymorphism of sequelae after COVID-19 in young adults." medRxiv (2022). | Wrong outcomes |
| Kanberg, Nelly, et al. "Neurochemical signs of astrocytic and neuronal injury in acute COVID-19 normalizes during long-term follow-up." EBioMedicine 70 (2021): 103512. | Wrong comparator |
| Kopishinskaia, Svetlana, et al. "Clinical features in russian patients with COVID-associated parosmia/phanthosmia." Psychiatria Danubina 33.suppl 9 (2021): 130-136. | Wrong study duration |
| Liampas, Andreas, et al. "Chronic pain in patients with COVID-19: Cross sectional study." (2021). | Wrong outcomes |
| Lin, MeiXuan, et al. "Health-related quality of life of COVID-19 survivors at 6 months after hospital discharge: a cohort study." (2021). | Wrong comparator |
| Liu, Yu-Hui, et al. "Post-infection cognitive impairments in a cohort of elderly patients with COVID-19." Molecular neurodegeneration 16.1 (2021): 1-10. | Wrong outcomes |
| Liu, Yu-Hui, et al. "Post-infection cognitive impairments in a cohort of elderly patients with COVID-19." (2021). | Conference abstract |
| Lund, Lars Christian, et al. "Post-acute effects of SARS-CoV-2 infection in individuals not requiring hospital admission: a Danish population-based cohort study." The Lancet Infectious Diseases 21.10 (2021): 1373-1382. | Wrong study duration |
| MacIntosh, Bradley J., et al. "Brain structure and function in people recovering from COVID-19 after hospital discharge or self-isolation: a longitudinal observational study protocol." Canadian Medical Association Open Access Journal 9.4 (2021): E1114-E1119. | Protocol |
| Magdy, Doaa M., et al. "Long-term COVID-19 effects on pulmonary function, exercise capacity, and health status." Annals of Thoracic Medicine 17.1 (2022): 28. | Wrong comparator |
| Magnúsdóttir, Ingibjörg, et al. "Acute COVID-19 severity and mental health morbidity trajectories in patient populations of six nations: an observational study." The Lancet Public Health 7.5 (2022): e406-e416. | Wrong outcomes |
| Marasco, Giovanni, et al. "Prevalence of gastrointestinal symptoms in severe acute respiratory syndrome coronavirus 2 infection: results of the prospective controlled multinational GI-COVID-19 study." The American Journal of Gastroenterology 117.1 (2022): 147-157. | Wrong outcomes |
| Murga, Iñigo, et al. "Clinical Heterogeneity in ME/CFS. A Way to Understand Long-COVID19 Fatigue." Frontiers in Psychiatry (2021): 1734. | Wrong population |
| Mutubuki, Elizabeth N., et al. "Prevalence and determinants of persistent symptoms after infection with SARS-CoV-2: Protocol for an observational cohort study (LongCOVID-study)." BMJ open 12.7 (2022): e062439. | Protocol |
| Nguyen, Nhu Ngoc, et al. "Long-term persistence of symptoms of dyspnoea in COVID-19 patients." International Journal of Infectious Diseases 115 (2022): 17-23. | Wrong study design |
| Oh, Tak Kyu, Hye Youn Park, and In‐Ae Song. "Risk of psychological sequelae among coronavirus disease‐2019 survivors: a nationwide cohort study in South Korea." Depression and Anxiety 38.2 (2021): 247-254. | Wrong outcomes |
| Oikonomou, Evangelos, et al. "Endothelial dysfunction in acute and long standing COVID− 19: A prospective cohort study." Vascular pharmacology 144 (2022): 106975. | Wrong outcomes |
| Paneroni, Mara, et al. "Muscle strength and physical performance in patients without previous disabilities recovering from COVID-19 pneumonia." American journal of physical medicine & rehabilitation 100.2 (2021): 105-109. | Wrong comparator |
| Guo, Panyuan, et al. "COVCOG 1: Factors predicting Cognitive Symptoms in Long COVID. A First Publication from the COVID and Cognition Study." medRxiv (2021). | Wrong study design |
| Phetsouphanh, Chansavath, et al. "Immunological dysfunction persists for 8 months following initial mild-to-moderate SARS-CoV-2 infection." Nature immunology 23.2 (2022): 210-216. | Wrong outcomes |
| Sandmann, Frank, et al. "Long-term health-related quality of life in non-hospitalised COVID-19 cases with confirmed SARS-CoV-2 infection in England: Longitudinal analysis and cross-sectional comparison with controls." medRxiv (2021). | Wrong outcomes |
| Daugherty, Sarah E., et al. "SARS-CoV-2 infection and risk of clinical sequelae during the post-acute phase: a retrospective cohort study." medRxiv (2021). | Wrong study duration |
| Siegerink, Sebastiaan, et al. "35. Health-related quality of life in COVID-19 survivors after 12 months, a prospective cohort study." Open Forum Infectious Diseases. Vol. 8. No. Supplement_1. US: Oxford University Press, 2021. | Wrong comparator |
| Stallmach, Andreas, et al. "Comparison of fatigue, cognitive dysfunction and psychological disorders in post-COVID patients and patients after sepsis: is there a specific constellation?." Infection (2022): 1-9. | Wrong comparator |
| Taquet, Maxime, et al. "6-month neurological and psychiatric outcomes in 236 379 survivors of COVID-19: a retrospective cohort study using electronic health records." The Lancet Psychiatry 8.5 (2021): 416-427. | Wrong outcomes |
| Wolfe, K. S., et al. "Functional and quality of life outcomes of critically ill covid-19 survivors at hospital discharge and six months." D9. D009 A BROADER VIEW OF OUTCOMES AFTER CRITICAL ILLNESS. American Thoracic Society, 2021. A1212-A1212. | Conference abstract |
| Woo, Marcel S., et al. "Frequent neurocognitive deficits after recovery from mild COVID-19." Brain communications 2.2 (2020): fcaa205. | Wrong study duration |
| Xiong, Qiutang, et al. "Clinical sequelae of COVID-19 survivors in Wuhan, China: a single-centre longitudinal study." Clinical Microbiology and Infection 27.1 (2021): 89-95. | Wrong outcomes |
| Yuan, Yuan, et al. "Prevalence of post-traumatic stress symptoms and its associations with quality of life, demographic and clinical characteristics in COVID-19 survivors during the post-COVID-19 era." Frontiers in Psychiatry 12 (2021): 665507. | Wrong outcomes |

## Table S4: Additional study/patient characteristics of included studies

| **Study** | **COVID+ period** | **Estimated variant at time of study** | **Female (%)** | **COVID+ average age or age range (years)** |
| --- | --- | --- | --- | --- |
| Al-Aly 2021^23^ | Mar 2020 to Nov 2020 | Wild type | 12.1% | Mean: 60.7 |
| Al-Aly 2022^22^ | Jan 2021 to Oct 2021 | Alpha, Delta | 14.9% | Mean: 66.6 |
| Amin-Chowdhury 2021^24^ | Mar 2020 to Apr 2020 | Wild type | 71.3% | Median: 41 |
| Borch 2022^25^ | Jan 2020 to Mar 2021 | Wild type | NR | Mean (0-5 years): 2.7  Mean (6-17): 12.0 |
| Carazo 2022^44^ | July 2020 to May 2021 | Wild type, Alpha | Hospitalised: 70.3%  Non-hospitalised: 79.2% | Mean (hospitalised): 46.7  Mean (non-hospitalised): 40.0 |
| Caspersen 2022^26^ | Mar 2020 to Jan 2021 | Wild type, Alpha | 58% | 25-34: 0.8%  35-39: 7.5%  40-44: 26.0%  45-49: 37.7%  50-54: 19.4%  55-59: 6.8%  60-64: 1.3%  65+: 0.5% |
| Castro 2021^27^ | Mar 2020 to May 2021 | Wild type, Alpha | 47% | Median: 63 |
| Chevinsky 2021^28^ | Mar 2020 to Jun 2020 | Wild type | 61.2% | 18-39: 35.7%  40-49: 18.1%  50-64: 25.9%  65-74: 10.3%  75-84: 6.0%  85+: 4.0% |
| Desgranges 2021^29^ | Feb 2020 to Apr 2020 | Wild type | 62% | Median: 41 |
| Elkan 2021^46^ | Mar 2020 to Jul 2020 | Wild type | 57.1% | Median: 42 |
| Huang 2021^47^ | Jan 2020 to May 2020 | Wild type | 46% | Median: 58 |
| Kikkenborg Berg 2022^30^ | Jan 2020 to Jul 2021 | Wild type, Alpha, Delta | 58.4% | Median: 17.6 |
| Kuodi 2022^31^ | Mar 2020 to Nov 2021 | Wild type, Alpha, Beta, Gamma, Delta | 62.3% | 19-35: 30.3%  36-60: 49.2%  >60: 20.5% |
| Liu 2022^45^ | Feb 2020 to Apr 2020 | Wild type | 52.0% | Median: 69 |
| Matta 2022^32^ | May 2020 to Nov 2020 | Wild type | 57.9% | Mean: 42.4 |
| Nielsen 2021^42^ | Mar 2020 to Jun 2020 | Wild type | 84.3% | <30: 15.7%  30–39: 23.3%  40–49: 30.5%  50–59: 23.3%  ≥60: 7.2% |
| Niyatiwatchanchai 2022^48^ | Apr 2021 to May 2021 | Alpha | 48.6% | Mean: 35.6 |
| Noviello 2022^33^ | Feb 2020 to Apr 2020 | Wild type | 40.2% | Mean: 44.1 |
| Petersen 2022^43^ | Mar 2020 to Dec 2020 | Wild type | 52.6% | Median: 55 |
| Radtke 2021^54^ | Oct 2020 to Nov 2020 | Wild type | 53% | 6-11: 61%  12-16: 39% |
| Raman 2020^49^ | Mar 2020 to May 2020 | Wild type | 41.4% | Mean: 55.4 |
| Rivera‑Izquierdo 2022^34^ | Mar 2020 to Apr 2020 | Wild type | 42.6% | Mean: 61.2 |
| Roessler 2021^35^ | Jan 2019 to Jun 2020 | Wild type | 59.3% | 0‐11: 5.1%  12‐17: 2.5%  18-24: 8.2%  25-39: 23.3%  40‐49: 15.8%  50‐54: 10.4%  55-59: 10.4%  60-64: 7.4%  65-69: 3.8%  70-74: 3.0%  74‐79: 2.9%  80+: 7.3% |
| Roge 2021^36^ | Jan 2020 to Mar 2021 | Wild type, Alpha | 44.5% | Median: 10 |
| Søras 2021a^51^ | Feb 2020 to Apr 2020 | Wild type | 55% | Mean: 47.3 |
| Søras 2021b^52^ | Feb 2020 to Apr 2020 | Wild type | 54% | Mean: 47.3 |
| Sørenson 2022^37^ | Sep 2020 to Apr 2021 | Wild type | 58.7% | Median: 49 |
| Spotnitz 2021^38^ | NR | NA | NR | NR |
| Stephenson 2022^53^ | Jan 2021 to Mar 2021 | Alpha* | 63.5% | 11-14: 40.6%  15-17: 59.4% |
| Strahm 2022^39^ | Jul 2020 to Mar 2021 | Wild type | 81% | Median: 38.9 |
| Taquet 2021^40^ | Jan 2020 to Dec 2020 | Wild type | 58.4% | Mean: 39.4 |
| Vlake 2021^50^ | Mar 2020 to Apr 2020 | Wild type | 36% | Median: 61 |
| Xie 2021^41^ | Mar 2020 to Mar 2021 | Wild type, Alpha | Non-hospitalised: 11.7% Hospitalised: 6.0% Intensive care): 5.4% | Median (non-hospitalised): 62.2 Median (hospitalised): 70.5  Median (intensive care): 70.8 |

* Reported by study

## Table S5: Risk of fatigue in COVID+ participants ≥4 weeks after infection compared to COVID- participants

| **Study** | **Fatigue description** | **N cases/controls** | **Time after infection** | **Risk measurement** | **Risk (95% CI)** |  |
| --- | --- | --- | --- | --- | --- | --- |
| **Hospitalised/outpatients only** | | | | | | |
| Al-Aly 2021^23^ | Malaise and fatigue | 11,287/795,025 | 30 days-6 months | Hazard ratio | 1.69 (1.59, 1.79) |  |
| Al-Aly 2022^22^ | Fatigue | 3,667/4,983,491 | 6 months | Hazard ratio | 4.49 (3.88, 5.19) |  |
|  |  | ICU: 811/4,983,491 | 6 months | Hazard ratio | 5.67 (4.28, 7.50) |  |
| **Castro 2021**^27^ | **Fatigue** | **6,619/6,342** | 31-90 days | Odds ratio | 0.98 (0.88, 1.10) |  |
|  |  |  | **91-150 days** | **Odds ratio** | **0.98 (0.84, 1.15)** |  |
| **Chevinsky 2021**^28^ | **Malaise and fatigue** | **44,489/44,489** | 31-60 days | Odds ratio | 1.5 (1.1, 1.8) |  |
|  |  |  | 61-90 days | Odds ratio | 0.84 (0.65, 1.1) |  |
|  |  |  | **91-120 days** | **Odds ratio** | **1.1 (0.90, 1.4)** |  |
| **Desgranges 2021**^29^ | **Fatigue** | **418/89** | **>3 months** | **Odds ratio** | **2.14 (1.04, 4.41)** |  |
| **Rivera‑Izquierdo 2022**^34^ | **Fatigue** | **453/453** | **12 months** | **Odds ratio** | **0.57 (0.36, 0.90)** |  |
| **Roessler 2021**^35^ | **Malaise/ fatigue/ exhaustion** | Children: 145,184/3,106,010 | 3 months | Incidence rate ratio | 1.97 (1.89, 2.06) |  |
|  |  | **Adults: 11,950/288,815** | **3 months** | **Incidence rate ratio** | **2.28 (1.71, 3.06)** |  |
| Roge 2021^36^ | Fatigue | 236/142 | 1-6 months | Odds ratio | 8.7 (2.5, 29.9) |  |
| **All COVID-19** | | | | | | |
| Al-Aly 2021^23^ | Malaise and fatigue | 68,984/4,818,281 | 30 days-6 months | Hazard ratio | 1.79 (1.65, 1.94) |  |
| **Al-Aly 2022**^22^ | **Fatigue** | **33,940/4,983,491** | **6 months** | **Hazard ratio** | **2.00 (1.82, 2.21)** |  |
|  |  |  | 30-90 days | Hazard ratio | 2.38 (2.13, 2.66) |  |
|  |  |  | 91-180 days | Hazard ratio | 1.54 (1.27, 1.88) |  |
|  |  | Non-hospitalised: 30,273/4,983,491 | 6 months | Hazard ratio | 1.34 (1.12, 1.61) |  |
|  | Incident fatigue | 33,940/4,983,491 | 91-180 days | Hazard ratio | 1.45 (1.16, 1.81) |  |
|  | Persistent fatigue | 33,940/4,983,491 | 91-180 days | Hazard ratio | 2.38 (1.62, 3.49) |  |
| **Amin-Chowdhury 2021**^24^ | **Unusual fatigue/tiredness after exertion** | **140/1,160** | **6 months** | **Odds ratio** | **2.72 (1.82, 4.07)** |  |
| Borch 2022^25^ | Fatigue | 14,883/15,234 | >4 weeks | Risk difference | 0.06 (0.06, 0.07) |  |
| **Caspersen 2022**^26^ | **Fatigue** | **116/2,222** | **1-6 months** | **Risk ratio** | **6.4 (5.4, 7.5)** |  |
|  |  | 29/2,634 | 11-12 months | Risk ratio | 4.8 (3.5, 6.7) |  |
| **Kikkenborg Berg 2022**^30^ | **Fatigue** | **5,106/21,640** | **2 months** | **Odds ratio** | **1.06 (0.98, 1.14)** |  |
| **Kuodi 2022**^31^ | **Fatigue** | **294/2,437** | **Mean 114.5 (IQR 340) days** | **Risk ratio** | **0.64 (0.46, 0.89)** |  |
| **Matta 2022**^32^ | **Fatigue** | **1,091/25,732** | **>4 weeks** | **Odds ratio** | **2.57 (2.00, 3.29)** |  |
| **Noviello 2022**^33^ | **Chronic fatigue** | **164/183** | **Mean 4.8 (SD 0.3) months** | **Risk ratio** | **2.24 (1.48, 3.37)** |  |
| Sørenson 2022^37^ | Fatigue/ exhaustion | 61,002/91,878 | 6-12 months | Risk difference | 0.08 (0.08, 0.09) |  |
|  |  |  | 6 months | Risk difference | 0.10 (0.09, 0.11) |  |
|  |  |  | 9 months | Risk difference | 0.08 (0.08, 0.09) |  |
|  |  |  | 12 months | Risk difference | 0.07 (0.06, 0.08) |  |
|  | Chronic fatigue syndrome | 61,002/91,878 | 6-12 months | Risk difference | 0.02 (0.02, 0.03) |  |
| **Strahm 2022**^39^ | **Fatigue** | **556/2550** | **>4 weeks** | **Odds ratio** | **2.6 (2.1, 3.3)** |  |
| **Xie 2021**^41^ | **General fatigue** | **181,384/181,384** | **6 months** | **Hazard ratio** | **1.75 (1.68, 1.81)** |  |
| **Hospitalised patients compared to historical influenza controls** | | | | | | |
| Al-Aly 2021^23^ | Malaise and fatigue | 11,287/11,401 | 30 days-6 months | Hazard ratio | 1.40 (1.28, 1.53) |  |
| Al-Aly 2022^22^ | Fatigue | 33,940/5,785,273 | 6 months | Hazard ratio | 1.46 (1.21, 1.76) |  |
| Spotnitz 2021^38^ | Malaise or fatigue | 448,176/803,870 | 30-180 days | Risk ratio | 1.30 (NR, NR) |  |
| Taquet 2021^40^ | Fatigue | 273,384/106,578 | 3-6 months | Absolute risk increase | 2.65 (2.22, 3.08) |  |

Data included in main meta-analysis indicated in bold

## Table S6: Risk of shortness of breath in COVID+ participants ≥4 weeks after infection compared to COVID- participants

| **Study** | **Shortness of breath description** | **N cases/controls** | **Time after infection** | **Risk measurement** | **Risk (95% CI)** |  |
| --- | --- | --- | --- | --- | --- | --- |
| **Hospitalised/outpatients only** | | | | | | |
| Al-Aly 2021^23^ | Shortness of breath | 11,287/795,025 | 30 days-6 months | Hazard ratio | 3.19 (2.88, 3.53) |  |
|  |  | ICU: 3586/NR | 30 days-6 months | Hazard ratio | 5.17 (4.57, 5.84) |  |
| Al-Aly 2022^22^ | Shortness of breath | 3,667/4,983,491 | 6 months | Hazard ratio | 3.54 (3.13, 4.00) |  |
|  |  | ICU: 811/4,983,491 | 6 months | Hazard ratio | 4.20 (3.13, 5.62) |  |
| **Rivera‑Izquierdo 2022**^34^ | **Dyspnoea** | **453/453** | **12 months** | **Odds ratio** | **1.13 (0.76, 1.68)** |  |
| **Desgranges 2021**^29^ | **Dyspnea** | **418/89** | **>3 months** | **Odds ratio** | **2.81 (1.10, 7.16)** |  |
|  | NYHA dyspnea class ≥2 | 418/89 | >3 months | Odds ratio | 1.11 (0.29, 4.32) |  |
| **Roessler 2021**^35^ | **Dyspnea** | **145,184/3,106,010** | **3 months** | **Incidence rate ratio** | **2.88 (2.74, 3.02)** |  |
| Roge 2021^36^ | Shortness of breath at rest | 236/142 | 1-6 months | Odds ratio | 2.1 (0.2, 18.4) |  |
| **All COVID-19** | | | | | | |
| **Al-Aly 2021**^23^ | **Shortness of breath** | **NR** | **30 days-6 months** | **Hazard ratio** | **1.72 (1.54, 1.92)** |  |
| **Al-Aly 2022**^22^ | **Shortness of breath** | **33,940/4,983,491** | **6 months** | **Hazard ratio** | **2.08 (1.95, 2.21)** |  |
|  |  | Non-hospitalised: 30,273/4,983,491 | 6 months | Hazard ratio | 1.91 (1.77, 2.06) |  |
| **Amin-Chowdhury 2021**^24^ | Breathlessness after minimal exertion | 140/1,160 | 6 months | Odds ratio | 3.06 (2.00, 4.67) |  |
|  | **Breathlessness at rest** | **140/1,160** | **6 months** | **Odds ratio** | **3.51 (1.68, 7.32)** |  |
| **Caspersen 2022**^26^ | **Shortness of breath** | **88/777** | **1-6 months** | **Risk ratio** | **13.9 (11.3, 17.1)** |  |
|  |  | 19/963 | 11-12 months | Risk ratio | 8.7 (5.7, 13.3) |  |
| **Kikkenborg Berg 2022**^30^ | **Trouble breathing** | **5,106/21,640** | **2 months** | **Odds ratio** | **2.70 (2.31, 3.15)** |  |
| **Kuodi 2022**^31^ | **Shortness of breath** | **294/2,437** | **Mean 114.5 (IQR 340) days** | **Risk ratio** | **1.52 (0.87, 2.65)** |  |
| **Matta 2022**^32^ | **Breathing difficulties** | **1,091/25,732** | **>4 weeks** | **Odds ratio** | **3.74 (2.57, 5.42)** |  |
| **Nielsen 2021**^42^ | **Dyspnea** | 181/581 | 31-60 days | Odds ratio | 6.76 (1.79, 25.47) |  |
|  |  | **148/515** | **61-90 days** | **Odds ratio** | **6.27 (0.53, 73.45)** |  |
| **Petersen 2022**^43^ | **NYHA dyspnea class 2** | **443/1,328** | **9 months** | **Odds ratio** | **1.10 (0.68, 1.79)** |  |
|  | NYHA dyspnea class 3 or 4 | 443/1,328 | 9 months | Odds ratio | 1.87 (1.06, 3.23) |  |
| Sørenson 2022^37^ | Dyspnea | 61,002/91,878 | 6-12 months | Risk difference | 0.05 (0.05, 0.05) |  |
|  |  |  | 6 months | Risk difference | 0.06 (0.05, 0.06) |  |
|  |  |  | 9 months | Risk difference | 0.05 (0.05, 0.05) |  |
|  |  |  | 12 months | Risk difference | 0.04 (0.04, 0.05) |  |
| **Hospitalised patients compared to historical influenza controls** | | | | | | |
| Al-Aly 2021^23^ | Shortness of breath | NR | 30 days-6 months | Hazard ratio | 1.14 (1.04, 1.26) |  |
| Al-Aly 2022^22^ | Shortness of breath | 33,940/5,785,273 | 6 months | Hazard ratio | 1.14 (0.94, 1.40) |  |
| Spotnitz 2021^38^ | Dyspnea | 448,176/803,870 | 30-180 days | Risk ratio | 2.28 (NR, NR) |  |

Data included in main meta-analysis indicated in bold

## Table S7: Risk of cognitive dysfunction in COVID+ participants ≥4 weeks after infection compared to COVID- participants

| **Study** | **Cognitive dysfunction description** | **N cases/controls** | **Time after infection** | **Risk measurement** | **Risk (95% CI)** |  |
| --- | --- | --- | --- | --- | --- | --- |
| **Hospitalised/outpatients only** | | | | | | |
| **Al-Aly 2022**^22^ | **Neurocognitive decline** | **3,667/4,983,491** | **6 months** | **Hazard ratio** | **3.06 (2.47, 3.79)** |  |
|  |  | ICU: 811/4,983,491 | 6 months | Hazard ratio | 3.7 (2.53, 5.41) |  |
| **Castro 2021**^27^ | **Impaired cognition** | **NR** | 31-90 days | Odds ratio | 0.89 (0.77, 1.02) |  |
|  |  |  | **91-150 days** | **Odds ratio** | **0.88 (0.71, 1.08)** |  |
|  | Memory complaints | NR | 31-90 days | Odds ratio | 0.86 (0.64, 1.14) |  |
|  |  |  | 91-150 days | Odds ratio | 0.84 (0.57, 1.20) |  |
|  | Language disturbance | NR | 31-90 days | Odds ratio | 0.42 (0.24, 0.68) |  |
|  |  |  | 91-150 days | Odds ratio | 0.56 (0.29, 1.00) |  |
| **Desgranges 2021**^29^ | **Memory impairment** | **418/89** | **>3 months** | **Odds ratio** | **5.71 (1.53, 21.3)** |  |
| **Liu 2022**^45^ | Early-onset cognitive decline | Non-severe: 260/438 | 6 months | Odds ratio | 1.71 (1.30, 2.27) |  |
|  |  | Severe: 1,178/438 | 6 months | Odds ratio | 4.87 (3.30, 7.20) |  |
|  | Late-onset cognitive decline | Non-severe: 260/438 | 6-12 months | Odds ratio | 1.59 (0.82, 3.09) |  |
|  |  | Severe: 1,178/438 | 6-12 months | Odds ratio | 7.58 (3.58, 16.03) |  |
|  | Progressive cognitive decline | Non-severe: 260/438 | 12 months | Odds ratio | 0.61 (0.27, 1.40) |  |
|  |  | Severe: 1,178/438 | 12 months | Odds ratio | 19.00 (9.14, 39.51) |  |
|  | **Cognitive impairment** | **Non-severe: 260/438** | **12 months** | **Odds ratio** | **1.10 (0.69, 1.76)** |  |
|  |  | Severe: 1,178/438 | 12 months | Odds ratio | 9.10 (5.61, 14.75) |  |
| **Rivera‑Izquierdo 2022**^34^ | **Confusion or memory loss** | **453/453** | **12 months** | **Odds ratio** | **1.83 (0.74, 4.81)** |  |
| Roge 2021^36^ | Difficulty concentrating | 236/142 | 1-6 months | Odds ratio | 13.6 (1.7, 105.3) |  |
|  | Impaired memory | 236/142 | 1-6 months | Odds ratio | 7.3 (0.9, 58.9) |  |
|  | Impaired attention | 236/142 | 1-6 months | Odds ratio | 19.0 (2.4, 147.5) |  |
| **All COVID-19** | | | | | | |
| Al-Aly 2022^22^ | Neurocognitive decline | 33,940 | 6 months | Hazard ratio | 1.70 (1.54, 1.88) |  |
|  | Neurocognitive decline | Non-hospitalised: 30,273/4,983,491 | 6 months | Hazard ratio | 1.45 (1.26, 1.66) |  |
| **Amin-Chowdhury 2021**^24^ | **Short-term memory loss** | **140/1,160** | **6 months** | **Odds ratio** | **5.66 (3.31, 9.67)** |  |
|  | **Confusion/brain fog/trouble focusing attention** | **140/1,160** | **6 months** | **Odds ratio** | **2.82 (1.81, 4.38)** |  |
|  | Forgetfulness | 140/1,160 | 6 months | Odds ratio | 2.62 (1.74, 3.95) |  |
|  | Trouble trying to form words | 140/1,160 | 6 months | Odds ratio | 2.24 (1.32, 3.81) |  |
| Borch 2022^25^ | Concentration difficulties | 14,883/15,234 | >4 weeks | Risk difference | -0.03 (-0.04, -0.03) |  |
| **Carazo 2022**^44^ | **Difficulty concentrating** | **6,061/4,390** | **4 weeks** | **Prevalence ratio** | **2.6 (2.4, 2.8)** |  |
|  | Difficulty organising oneself | 6,061/4,390 | 4 weeks | Prevalence ratio | 2.8 (2.5, 3.1) |  |
|  | **Forgetfulness** | **6,061/4,390** | **4 weeks** | **Prevalence ratio** | **3.0 (2.6, 3.4)** |  |
|  | Losing necessary items | 6,061/4,390 | 4 weeks | Prevalence ratio | 2.2 (1.9, 2.7) |  |
| **Caspersen 2022**^26^ | **Poor memory** | **81/1,798** | **1-6 months** | **Risk ratio** | **5.6 (4.5, 6.8)** |  |
|  |  | 30/2,517 | 11-12 months | Risk ratio | 5.3 (3.8, 7.3) |  |
|  | **Brain fog** | **84/2,227** | **1-6 months** | **Risk ratio** | **4.7 (3.8, 5.7)** |  |
|  |  | 20/2,780 | 11-12 months | Risk ratio | 3.2 (2.1, 4.8) |  |
| **Kikkenborg Berg 2022**^30^ | **Trouble remembering or concentrating** | **5,106/21,640** | **2 months** | **Odds ratio** | **1.04 (0.97, 1.12)** |  |
| **Kuodi 2022**^31^ | **Loss of concentration** | **294/2,437** | **Mean 114.5 (IQR 340) days** | **Risk ratio** | **1.219 (0.690, 2.154)** |  |
| **Matta 2022**^32^ | **Poor attention or concentration** | **1,091/25,732** | **>4 weeks** | **Odds ratio** | **2.10 (1.56, 2.82)** |  |
| Sørenson 2022^37^ | Difficulties concentrating | 61,002/91,878 | 6-12 months | Risk difference | 0.28 (0.28, 0.29) |  |
|  | Memory issues | 61,002/91,878 | 6-12 months | Risk difference | 0.27 (0.26, 0.27) |  |
| **Hospitalised patients compared to historical influenza controls** | | | | | | |
| Al-Aly 2022^22^ | Neurocognitive decline | 33,940/5,785,273 | 6 months | Hazard ratio | 1.47 (1.15, 1.87) |  |
| Spotnitz 2021^38^ | Dementia or cognitive impairment | 448,176/803,870 | 30-180 days | Risk ratio | 1.35 (NR, NR) |  |
| Taquet 2021^40^ | Mild cognitive impairment | 273,384/106,578 | 3-6 months | Absolute risk increase | 1.18 (0.89, 1.48) |  |

Data included in main meta-analysis indicated in bold

## Table S8: Quality of life reported by the included studies

| **Study** | **Population** |  | | **QoL instrument** | **QoL summary for COVID-19 cases** |  |
| --- | --- | --- | --- | --- | --- | --- |
| **Hospitalised COVID-19** | | |  |  |  |  |
| Elkan 2021^46^ | Adults | Median 7.5 months (IQR 6-9) | | RAND-36 | Significantly worse self-perceived health compared to one year ago and no difference in any other domain compared to hospitalised pneumonia patients |  |
| Huang 2021^47^ | Adults | 12 months | | EQ-5D | Significantly worse QoL in nearly all domains compared to healthy controls |  |
| Niyatiwatchanchai 2022^48^ | Adults | 1 month | | EQ-5D, SF-36 | Significantly worse QoL in almost all domains for severe COVID-19 and significantly worse in some domains for non-severe compared to healthy controls |  |
| Raman 2020^49^ | Adults | 2-3 months | | SF-36 | Significantly worse QoL in all domains compared to healthy controls |  |
| Vlake 2021^50^ | Adults | 1 month, 3 months | | EQ-5D, RAND-36 | No significant difference at 3 months compared to non-COVID-19 hospitalisations, and only significantly worse physical domain scores at 1 month |  |
| **All COVID-19** | | | | | | |
| Kikkenborg Berg 2022^30^ | Adolescents (15-18 yrs) | 2-12 months | | CSSI-24, PedsQL | Significantly better QoL in both instruments |  |
| Petersen 2022^43^ | Adults (45-74 yrs) | 9 months | | EQ-5D | No significant difference |  |
| Radtke 2021^54^ | Children and adolescents (6-16 yrs) | ≥4 weeks | | HBSC | No significant difference |  |
| Søras 2021a^51^ | Adults | 3 months | | RAND-36 | A larger percentage reported worse health compared to one year ago |  |
| Søras 2021b^52^ | Adults | 8 months | | RAND-36 | A larger percentage reported worse health compared to one year ago |  |
| Stephenson 2022^53^ | Adolescents (11-17 yrs) | 3 months | | EQ-5D | 11-14 yrs were more likely to report physical problems, and 15-17 yrs were more likely to report mental problems |  |

CSSI-24, Children’s Somatic Symptoms Inventory-24; EQ-5D, EuroQol-5D; HBSC, Health Behaviour in School-aged Children; PedsQL, Paediatric Quality of Life Inventory; SF-36, 36-Item Short Form Survey.

## Table S9: Quality assessment scores of included studies

| Author/year | Selection  (max 4 stars) | Comparability (max 2 stars) | Outcome  (max 3 stars) | Total  (out of 9 stars) | Risk of bias |
| --- | --- | --- | --- | --- | --- |
| Al-Aly 2021^23^ | 2 | 2 | 3 | 7 | Low |
| Al-Aly 2022^22^ | 3 | 2 | 3 | 8 | Low |
| Amin-Chowdhury 2021^24^ | 3 | 2 | 2 | 7 | Low |
| Borch 2022^25^ | 3 | 0 | 2 | 5 | Medium |
| Carazo 2022^44^ | 3 | 2 | 2 | 7 | Low |
| Caspersen 2022^26^ | 3 | 2 | 2 | 7 | Low |
| Castro 2021^27^ | 2 | 2 | 3 | 7 | Low |
| Chevinsky 2021^28^ | 3 | 2 | 3 | 8 | Low |
| Desgranges 2021^29^ | 4 | 2 | 3 | 9 | Low |
| Elkan 2021^46^ | 3 | 1 | 2 | 6 | Medium |
| Huang 2021^47^ | 4 | 2 | 2 | 8 | Low |
| Kikkenborg Berg 2022^30^ | 4 | 1 | 2 | 7 | Low |
| Kuodi 2022^31^ | 3 | 1 | 0 | 4 | Medium |
| Liu 2022^45^ | 3 | 2 | 3 | 8 | Low |
| Matta 2022^32^ | 3 | 2 | 1 | 6 | Medium |
| Nielsen 2021^42^ | 3 | 2 | 1 | 6 | Medium |
| Niyatiwatchanchai 2022^48^ | 3 | 2 | 3 | 8 | Low |
| Noviello 2022^33^ | 3 | 2 | 1 | 6 | Medium |
| Petersen 2022^43^ | 4 | 2 | 2 | 8 | Low |
| Radtke 2021^54^ | 4 | 0 | 1 | 5 | Medium |
| Raman 2020^49^ | 4 | 2 | 2 | 8 | Low |
| Rivera‑Izquierdo 2022^34^ | 2 | 2 | 2 | 6 | Medium |
| Roessler 2021^35^ | 3 | 2 | 3 | 8 | Low |
| Roge 2021^36^ | 3 | 1 | 2 | 6 | Medium |
| Søras 2021a^51^ | 4 | 2 | 1 | 7 | Low |
| Søras 2021b^52^ | 3 | 2 | 2 | 7 | Low |
| Sørenson 2022^37^ | 2 | 2 | 2 | 6 | Medium |
| Spotnitz 2021^38^ | 2 | 0 | 2 | 4 | Medium |
| Stephenson 2022^53^ | 4 | 2 | 2 | 8 | Low |
| Strahm 2022^39^ | 3 | 2 | 2 | 7 | Low |
| Taquet 2021^40^ | 3 | 2 | 3 | 8 | Low |
| Vlake 2021^50^ | 1 | 2 | 2 | 5 | Medium |
| Xie 2021^41^ | 2 | 2 | 3 | 7 | Low |
